# 3’UTR shortening of HAS2 promotes hyaluronan hyper-synthesis and bioenergetic dysfunction in pulmonary hypertension

**DOI:** 10.1016/j.matbio.2022.06.001

**Published:** 2022-06-04

**Authors:** Victor Tseng, Scott D. Collum, Ayed Allawzi, Kathryn Crotty, Samantha Yeligar, Aaron Trammell, M. Ryan Smith, Bum-Yong Kang, Roy L. Sutliff, Jennifer L. Ingram, Soma S.S.K. Jyothula, Rajarajan A. Thandavarayan, Howard J. Huang, Eva S. Nozik, Eric J. Wagner, C. Michael Hart, Harry Karmouty-Quintana

**Affiliations:** a **Respiratory Medicine,** Ansible Health, Mountain, View, CA; b **Department of Biochemistry and Molecular Biology,** McGovern Medical School, University of Texas Health Science Center at Houston, Houston, TX; c **Translate Bio,** Lexington, MA; d **Emory University Division of Pulmonary,** Allergy, Critical Care, and Sleep Medicine, Atlanta, GA; e **Atlanta Veteran Affairs Health Care System,** Decatur, GA; f **Duke University Department of Medicine,** Durham, NC; g **Divisions of Critical Care, Pulmonary & Sleep Medicine,** McGovern Medical School, University of Texas Health Science Center at Houston, Houston, TX; h **Debakey Heart & Vascular Center,** Houston Methodist Hospital, Houston, TX, USA; i **University of Colorado Anschutz Medical Campus,** Department of Pediatrics, Aurora, CO; j **University of Rochester Medical Center,** School of Medicine and Dentistry, Rochester, NY

**Keywords:** Pulmonary hypertensionVascular, biologyHyaluronanextracellular matrixmetabolismsmooth, muscle cellRNA processing deficiencyhypoxia

## Abstract

Pulmonary hypertension (PH) comprises a diverse group of disorders that share a common pathway of pulmonary vascular remodeling leading to right ventricular failure. Development of anti-remodeling strategies is an emerging frontier in PH therapeutics that requires a greater understanding of the interactions between vascular wall cells and their extracellular matrices. The ubiquitous matrix glycan, hyaluronan (HA), is markedly elevated in lungs from patients and experimental models with PH. Herein, we identified HA synthase-2 (HAS2) in the pulmonary artery smooth muscle cell (PASMC) layer as a predominant locus of HA dysregulation. HA upregulation involves depletion of NUDT21, a master regulator of alternative polyadenylation, resulting in 3’UTR shortening and hyper-expression of HAS2. The ensuing increase of HAS2 and hyper-synthesis of HA promoted bioenergetic dysfunction of PASMC characterized by impaired mitochondrial oxidative capacity and a glycolytic shift. The resulting HA accumulation stimulated pro-remodeling phenotypes such as cell proliferation, migration, apoptosis-resistance, and stimulated pulmonary artery contractility. Transgenic mice, mimicking HAS2 hyper-synthesis in smooth muscle cells, developed spontaneous PH, whereas targeted deletion of HAS2 prevented experimental PH. Pharmacological blockade of HAS2 restored normal bioenergetics in PASMC, ameliorated cell remodeling phenotypes, and reversed experimental PH *in vivo*. In summary, our results uncover a novel mechanism of HA hyper-synthesis and downstream effects on pulmonary vascular cell metabolism and remodeling.

## INTRODUCTION

Pulmonary hypertension (PH) comprises a diverse group of disorders that converge on a final common pathway of increased vascular remodeling in the lung that leads to progressive right ventricular failure and death. Classification of PH is based on whether the pulmonary vascular changes are explained by pathology of the arteries themselves, left heart disease, chronic lung disease, recurrent thromboembolism, or a variety of systemic disorders (World Health Organization [WHO] Groups 1 - 5, respectively). Regardless of the underlying etiology, the pathogenesis of PH involves a combination of pulmonary vasoconstriction and pulmonary vascular remodeling. The remodeling process is characterized by hyperproliferative and apoptosis-resistant pulmonary vascular cell phenotypes, culminating in narrowing of the lumen and obstruction of blood flow.

Clinical outcomes in PH remain unacceptably poor, with median survival of only 3 years from time of diagnosis [1]. Available pulmonary vasodilator treatments have negligible anti-remodeling activity and are only indicated for a small minority of PH patients. Furthermore, vasodilator therapies become less effective in later stages of disease, when irreversible occlusive remodeling is the primary contributor to vascular resistance [2]. Consequently, there is an urgent need to identify and target novel pathways in PH that can reverse remodeling and pathobiology for the majority of patients suffering from this disorder.

The extracellular matrix (ECM) plays a crucial role in pulmonary vascular remodeling [3]. In addition to mechanical stiffening and abnormal vasomotor function of the pulmonary vessels, the ECM also directs pathologic cellular phenotypes such as proliferation and apoptosis-resistance. Increased deposition of hyaluronan (HA), the most abundant ECM glycosaminoglycan in the lung, has been reported in patients with various forms of PH due to an array of underlying conditions [4-8]. HA is synthesized by three synthase isoforms (HAS 1, 2, or 3) [9] and can be released from many cell types [10] and it has complex pleiotropic functions [11]. HA is also unique to other glycosaminoglycans in that it is not produced in the Golgi apparatus but instead is synthesized at the plasma membrane by HAS (1-3) [12]. HA modulates cellular secretion, proliferation and migration in a paracrine or autocrine manner [13]. Moreover, cleavage of large HA multimers by hyaluronidases generate low molecular weight fragments which participate in inflammatory [14], angiogenic [15] and proliferative responses [16] which are collectively implicated in PH. These versatile properties of HA lend biological plausibility for its potential role in vascular remodeling.

Recent evidence suggests that pathologic changes in the ECM play a critical role in provoking metabolic dysfunction [3,17,18]. ECM-driven metabolic reprograming of vascular wall cells is hypothesized to be a key contributor to pro-remodeling phenotypes such as proliferation, migration, and apoptosis-resistance. Our group has investigated upstream mechanisms of abnormal ECM production from mesenchymal cells. We previously identified alternative polyadenylation and resultant 3’UTR shortening of several ECM transcripts in dermal and pulmonary fibrosis [19,20]. The master regulator of polyadenylation, nudix hydrolase-21 (NUDT21, or cleavage factor Im subunit 25) maintains distal polyadenylation sites. Loss of NUDT21 leads to 3’UTR shortening, enhanced expression of profibrotic ECM genes, and enhanced tumor growth [21-24]. Taken together, these observations suggest that loss of NUDT21 and subsequent 3’UTR shortening may play an important role in PH by promoting cell proliferation or enhanced ECM production.

This study addresses the causes and consequences of HAS2 upregulation in PH. Specifically, it establishes that loss of NUDT21 in the pulmonary vasculature promotes 3’UTR shortening of HAS2 to drive increased HA production. Next, we show that HAS2 upregulation and HA hyper-synthesis promote metabolic reprogramming of vascular smooth muscle cells and stimulate pro-remodeling phenotypes. Finally, the therapeutic potential of HAS2 inhibition is tested in several distinct rodent models of PH. Our results establish loss of NUDT21 and 3’UTR shortening of HAS2 as a central axis in PH pathogenesis. We also identify inhibition of HAS2 as a promising anti-remodeling therapeutic approach for PH.

## METHODS

A detailed description of materials, reagents, and experimental protocols is provided in the Online Supplemental File.

### Ethics Statement

Explanted de-identified lung tissue from patients with idiopathic pulmonary arterial hypertension and corresponding untraceable clinical data were obtained from the Methodist Hospital (Houston, TX) as described previously [25]. Failed donor control lungs were obtained from the International Institute for the Advancement of Medicine (Edison, NJ). All human tissues were obtained with informed consent. The study conformed to the principles asserted in the Declaration of Helsinki. The study was reviewed by the University of Texas Health Science Center Committee for the Protection of Human Subjects (Institutional Review Board no. HSC-MS-08-0354 and HSC-MS-15-1049).

### Rodent Studies

All animal protocols were approved by the Institutional Animal Care and Use Committee (IACUC) according to the guidelines established by the international Association for Assessment and Accreditation of Laboratory Animal Care (AAALAC, ‘Guide for the Care and Use of Laboratory Animals’, National Academies Press 2011). For right heart catheterization, mice and rats were anesthetized with continuous inhalation of 1.5 – 2.0% isoflurane under a nosecone. The rodents were euthanized in a carbon dioxide chamber followed by cervical dislocation and bilateral thoracotomy, in accordance with protocols established by the American Veterinary Medical Association (AVMA POE 2020 Guidelines).

### Data and Statistical Procedures

Data were analyzed with Prism 8 software (Graph-Pad; La Jolla, CA). For all rodent experiments, we used 8-12 independent animals per group, affording 80% power to exclude an effect size of at least 1.6 assuming a Gaussian distribution (α=0.05). All investigators were blinded to the rodent genotype, which was retroactively assigned by genotyping at the time of statistical analysis. *In vitro* experiments were carried out with between 4 and 6 unique cell lines on at least two separate occasions. For factorial design experiments with more than two groups, we utilized parametric 2-way ANOVA testing with Benjamini-Krieger-Yekutieli (BKY) *post hoc* test to control the false discovery rate. For unpaired data consisting of two groups, we utilized the parametric 2-tailed Welch’s *t*-test, accounting for unequal variances and/or sample sizes. For paired data consisting of repeated measures on the same group, we utilized parametric 2-tailed student’s paired *t*-test assuming uniform variances. For data pertaining to repeated observations of the same group subjects across time, such as before and after treatment, we utilized 1-way repeated measures ANOVA. Statistical significance was defined as p<0.05. Outliers were excluded only if they fulfilled the ROUT test and are reported in the corresponding figure legend whenever applicable.

## RESULTS

### Pulmonary vascular HA and HAS2 are pathologically elevated in PH

**Figure 1.**
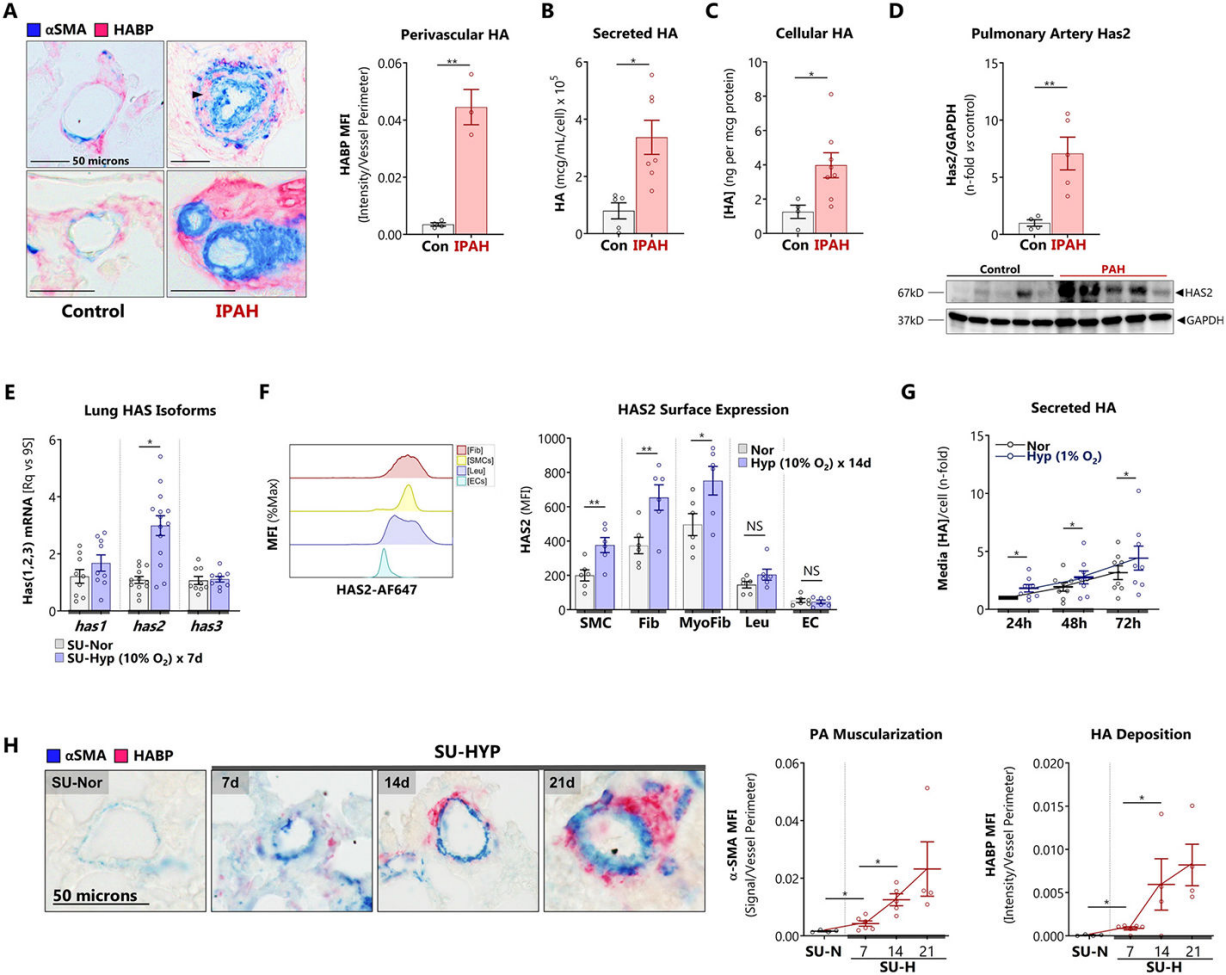
HA is pathologically elevated in human and experimental PH. A: Immunohistology for HA and αSMA was performed in explanted lungs from patients undergoing transplantation for end-stage PH versus failed donor controls. The perivascular HA (pink) was quantified. Top panels: small (50 – 100 μm) pulmonary arteries. Bottom panels: pre-arteriolar arteries (< 50 μm). Black arrowhead indicates intramedial HA staining. Scale bar: 50 μm. B – C: Levels of (B) secreted HA, (C) cell-bound HA, and were quantified in HPAMSCs (n = 5 – 7) isolated from distal vessels of patients undergoing transplantation for IPAH. D: Immunoblot for HAS2 protein isolated from control and PAH -derived pulmonary arteries (PAs), and subsequent densitometries. E: Three HAS isoforms were analyzed with RT-qPCR on whole lungs of mice with SU-HYP PH. F: Surface expression of HAS2 in the hypoxic mouse PH model was measured by flow cytometry. G: Cumulative HA production was measured by ELISA in the resampled supernatants of confluent HPASMCs cultured for 24, 48, and 72 hours in normoxic and hypoxic (1% O_2_) conditions. H: The time course of αSMA and HA deposition in pulmonary arteries ≤ 50 μm were elucidated in mice (n = 4 – 5) with SU-HYP induced PH. Data are expressed as mean ± SEM. *p < 0.05 and **p < 0.01 by Welch’s unpaired *t*-test (A – F), paired *t*-test (G), and 1-way ANOVA by time point (H). In (D), one control sample was removed from analysis as it fulfilled the ROUT and Grubb’s test as an outlier.

Consistent with previous reports, we confirmed that vascular HA was increased in explanted lungs of human subjects who underwent lung transplantation for end-stage IPAH (Figure 1A) [4,8,26]. Increased intra-medial HA staining between smooth muscle cell layers was apparent, suggesting potential elevated smooth muscle HA production. Indeed, we found that primary PASMCs isolated from patients with IPAH retained their HA hypersecretory phenotype *ex vivo.* Compared to controls, IPAH PASMC were characterized by elevated levels of secreted (Figure 1B) and cell-bound (Figure 1C) HA. Additionally, HAS2 protein levels were elevated in isolated pulmonary arteries (PA) from explanted IPAH lungs compared to failed donor control specimens (Figure 1D).

Next, we measured the expression of HA synthases in the Sugen-Hypoxia (SU-HYP) mouse model of PH. Hypoxic induction of *has2* mRNA was evident following 7 days of hypoxia exposure (Figure 1E). HAS2 is the most abundant HA synthase isoform in the lung [27] and is responsible for the synthesis of high molecular weight HA, contributing to the majority of HA content. To examine the surface expression of HAS2, we performed flow cytometry on lungs from SU-HYP mice. We found that smooth muscle cells, myofibroblasts, and fibroblasts but not leucocytes or endothelial cells demonstrated hypoxic upre3gulation of surface HAS2 (Figure 1F). Cultured HPASMCs were identified as major secretors of HA, producing levels comparable to fibroblasts, and uniquely displayed time-dependent hypoxia-inducible HA production (Figure 1G). Other cultured lung cell types did not demonstrate hypoxia-inducible HA secretion (Supplemental Figure S1A, B). Finally, we evaluated the spatial and temporal dynamics of HA accumulation *in vivo* with a histologic time course in the mouse hypoxia PH model. Pulmonary vascular HA was observed as early as day 7 and 14 in the SU-HYP model (Figure 1H). These data identify PASMC as a critical source of HA/HAS2 dysregulation and overproduction. However, the mechanisms that lead to upregulation of HAS2/HA in PH where hypoxia is an important molecular stimulus is not fully understood.

### NUDT21 depletion and 3’UTR shortening contributes to post-transcriptional upregulation of HAS2

We investigated potential mechanisms of HAS2 upregulation based on our recent demonstration that dysregulation of mRNA processing resulting from depletion of NUDT21 leads to 3’UTR shortening of genes involved in extracellular matrix remodeling [20]. The cleavage and polyadenylation specificity factor NUDT21 (CFIm25) regulates 3’UTR length [21]. Thus, we reasoned that NUDT21-related 3’UTR shortening of *has2* may account for increased expression of HAS2 and subsequent hyper-synthesis of HA.

**Figure 2.**
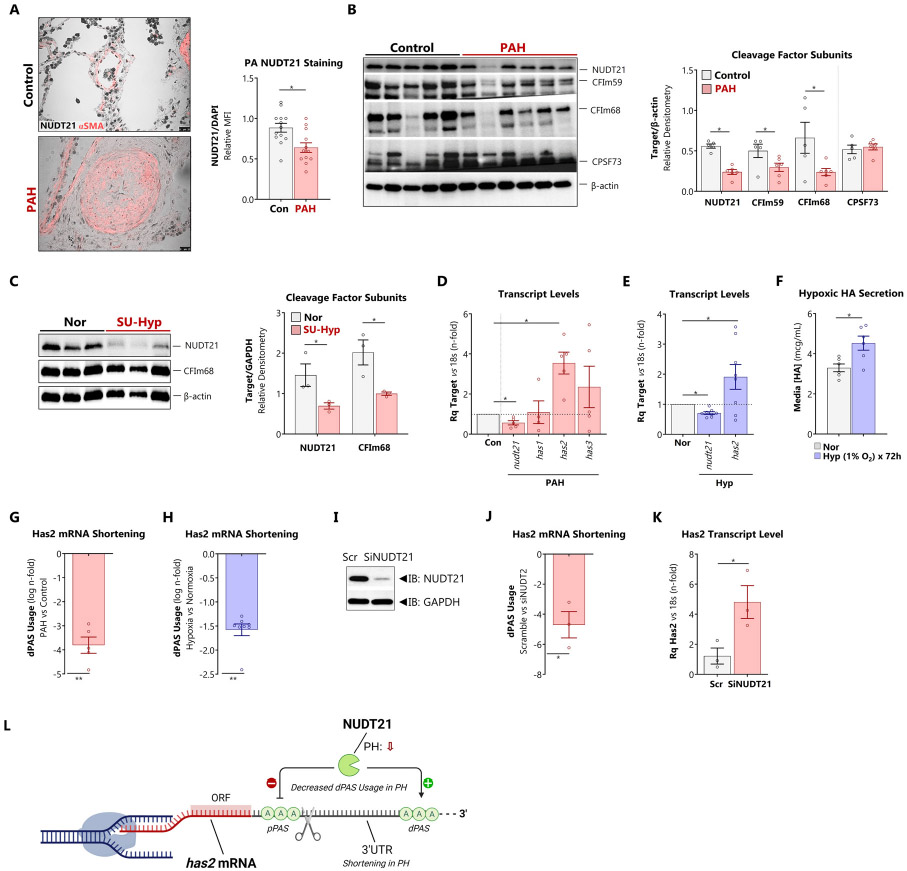
NUDT21 depletion and 3’UTR shortening contributes to post-transcriptional upregulation of HAS2 A: Explanted lungs from patients with PAH were co-stained for αSMA (pink)and NUDT21 (black). The sequential section was stained with DAPI (not shown). NUDT1/DAPI ratio was assessed by relative fluorescent intensity in αSMA-positive cells. B: Immunoblot from lysates prepared from isolated pulmonary arteries from patients with PAH versus failed donor controls. Expression of polyadenylation and cleavage factor subunits NUDT21, CFIm59, CFIm69, and CPFS73 was quantified as relative densitometry against β-actin. C: Immunoblot from lung lysates prepared from mice with SU-HYP-induced PH versus normoxic controls. Expression of NUDT21 and CFIm68 was quantified as relative densitometry against β-actin. D, G: (D) mRNA expression of NUDT21, HAS1, HAS2 and HAS3 and (G) HAS2 dPAS usage were quantified by PCR in explanted pulmonary arteries from control vs PAH patients. E, H: (E) mRNA expression of NUDT21 and HAS2 and (H) HAS2 dPAS usage were quantified by PCR in isolated human PASMCs exposed to normoxia vs hypoxia (1% O_2_ for 72 hours). F: Secreted HA content from isolated PASMCs under normoxic or hypoxic conditions, quantified by ELISA. I: Effective knockdown of NUDT21 by siRNA J, K: (J) HAS2 dPAS usage and (K) HAS2 mRNA abundance were determined in isolated human PASMCs depleted of NUDT21 with siRNA L: Schematic depiction of relationship between NUDT21, alternative polyadenylation, and Has2 3’UTR shortening. Data are expressed as mean ± SEM.> *p < 0.05 and **p < 0.01 by Welch’s unpaired *t*-test (A – E, G – K) or paired t-test (F).

We first determined expression levels of NUDT21 in PAH. Nuclear NUDT21 staining was reduced in remodeled vessels from patients with PAH (Figure 2A). Accordingly, NUDT21 protein expression was reduced in isolated PAs from patients with PAH compared to control (Figure 2B). NUDT21 trimerizes with CFIm59 and CFim68 to form Cleavage Factor Im (CFIm); both of these associated subunits were also depleted in isolated PAs (Figure 2B). The cleavage and polyadenylation specificity factor-73 (CPSF73), a critical subunit of the terminal pre-mRNA polyadenylation sequence recognition and processing assembly that does not complex with NUDT21, is preserved in PH, suggesting a unique role for the depletion of CFIm in PH. Next, we determined whether NUDT21 was suppressed in our SU-HYP mouse model of PH. These studies reveal approximately 50% loss of NUDT21 in lung tissue following 4 weeks of SU-HYP exposure (Figure 2C).

Expression profiles from isolated pulmonary arteries of PAH subjects show reduced NUDT21 mRNA levels, concomitant with *has2,* but not *has1* or *has3* (Figure 2D), suggesting a link between NUDT21 depletion and HAS2 expression. Next, to determine whether hypoxia plays a role in the depletion of NUDT21, we exposed HPASMCs to 1% O_2_ for 72 hours. Hypoxia led to a reduction in *nudt21* mRNA and upregulation of *has2* (Figure 2E), accompanied by increased secretion of HA (Figure 2F).

Following loss of NUDT21, alternative polyadenylation favors proximal sites, resulting in 3’UTR shortening [20,21]. We evaluated whether 3’UTR shortening affects HAS2 using dPAS-spanning PCR primers [20,21]. In PAH pulmonary arteries, dPAS-spanning primers revealed an ensuing decrease in *has2* dPAS usage indicating 3’ mRNA shortening (Figure 2G). Hypoxia exposure similarly promoted 3’UTR shortening in HPASMCs (Figure 2H). To confirm that NUDT21 was responsible for mediating HAS2 3’UTR shortening, NUDT21 was knocked down with siRNA in HPASMCs (Figure 2I). Silencing NUDT21 led to increased expression of HAS2 concomitant with 3’UTR shortening of the HAS2 transcript (Figure 2J and K). Collectively, these findings support that PH involves specific disruption of NUDT21-*has2* interaction rather than disruption of other components of polyadenylation machinery or other HA synthases. In summary, loss of NUDT21 and its associated cleavage complex subunits promotes 3’UTR shortening of *has2* leading to its upregulation and elevated production of HA (Figure 2L).

### Excessive HA synthesis induces mitochondrial dysfunction and glycolysis in HPASMCs

Metabolic reprograming in PH is increasingly recognized as a key contributor to pro-remodeling phenotypes of pulmonary vascular wall cells, such as proliferation, migration, and apoptosis-resistance. Recent evidence suggests that pathologic changes in the ECM play a critical role in provoking metabolic dysfunction [3,17,18]. We examined the bioenergetic consequences of HA in later stages of PH, characterized by high levels of extracellular HA. We used the Seahorse XF platform to analyze oxygen consumption rate (OCR) and extracellular acidification rate (ECAR) when HA levels were manipulated in HPASMCs.

**Figure 3.**
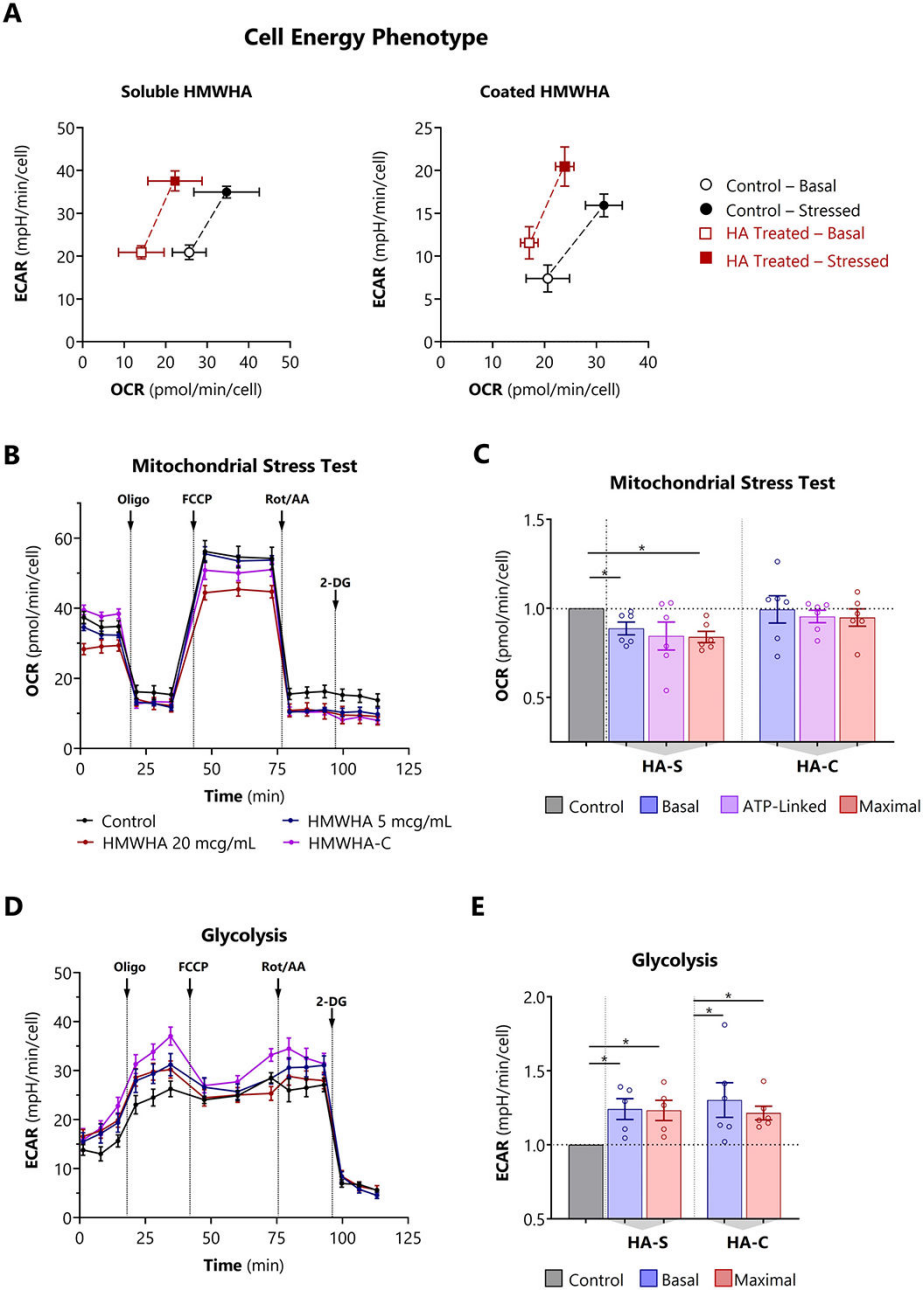
Excessive HA synthesis induces mitochondrial dysfunction and glycolysis in HPASMCs A: Cell energy phenotype tests performed by adding a stress mixture of oligomycin and FCCP concurrently using soluble HMWHA and coated HMWHA. B, D: Representative (B) OCR and (D) extracellular acidification rate (ECAR) profiles in cells treated with polydisperse HMWHA (average MW 1.5 megadaltons) for 72 hours or cultured on a HMWHA-coated (HA-C) surface. C, E: Determination of (C) including basal, ATP-linked, and maximal mitochondrial respiration, and (F) basal and maximal glycolysis from OCR and ECAR profiles respectively. Data expressed as mean ± 95% CI (A) or mean ± SEM (B – E). *p < 0.05 by 1-way ANOVA for each of the bioenergetic indices by treatment condition (C, E). In all bioenergetic experiments, OCR and ECAR are normalized to cell number per well. Comprehensive bioenergetic indices are presented in Supplemental Figure S2, and bioenergetic index definitions are summarized in Supplemental Figure S7.

First, cell energy phenotype testing was used to screen for qualitative metabolic switching in HPASCMs either exposed to exogenous soluble HMWHA spiked into the media (HA-S), or grown on HMWHA-coated polystyrene (HA-C). Both HA-S and HA-C reduced aerobic respiration, and HA-C also stimulated glycolysis (Figure 3A). Next, formal mitochondrial stress testing was performed to verify and further investigate the abnormal cell energy response. HA-S suppressed all key indices of mitochondrial oxidative respiration including basal, ATP-linked, maximal respiration (Figures 3B and 3C), and spare respiratory capacity. The suppressive effect was dose-dependent (Supplemental Figure S2A-D). At the same time, soluble HA-S also increased the ECAR, indicating heightened basal and maximal glycolysis (Figures 3 C-E). When HA was provisioned as a substrate coating, OCR was unchanged but glycolysis was increased.

Although we primarily examined HAS overexpression with high levels of exogenous HA, the equilibrium between HAS and HYAL activity appears to have a major influence on HPASMC bioenergetics. In rodent models of PH [28-30], there is early hyperdynamic turnover of perivascular HA which occurs *prior* to the onset of vascular remodeling and hemodynamic abnormalities. This turnover is due to simultaneous upregulation of HA synthesis and HA digestion, mediated primarily by HAS2 and HYAL2, respectively. Cyclic expansion and breakdown of pericellular HA facilitates migration and proliferation of stromal cells [31]. We modeled this early stage of acute HA recycling by treating HPASMCs simultaneously with Ad^CMV^-HAS2 and recombinant HYAL. Whereas neither HAS nor HYAL alone had significant effects on OCR or ECAR, combined HAS plus HYAL induced a highly aerobic phenotypes, with a 45% increase in basal, ATP-linked, and maximal oxygen consumption (Supplemental Figure S3).

Taken together, these findings are consistent with an HA-induced aerobic-glycolytic shift in HPASMCs (Warburg effect), a central feature of metabolic reprogramming observed in PH [32-34]. Furthermore, HA-driven bioenergetic changes were dependent on whether contact occurred primarily on basolateral or apical surfaces, and on the balance between HA synthesis and degradation.

### Excessive HA synthesis promotes HPASMC pro-remodeling phenotypes

PH involves a combination of occlusive vascular remodeling and abnormal vasomotor function. Therefore, we examined the impact of HA on HPASMC remodeling phenotypes *in vitro,* such as proliferation rate, migration, and apoptosis-resistance. In parallel, we evaluated the vasomotor effects of HA on pulmonary artery contractility and relaxation *ex vivo.*

**Figure 4.**
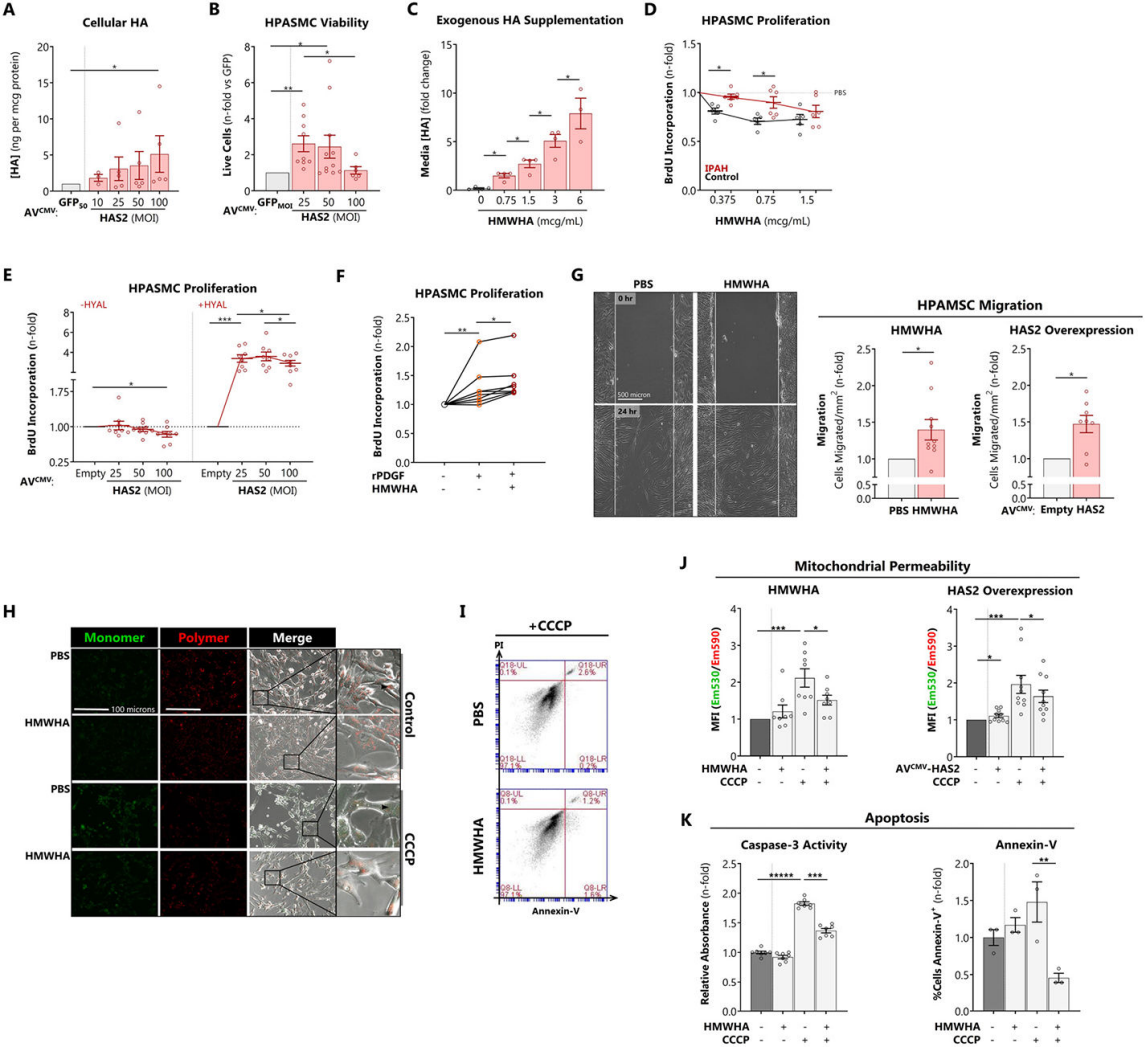
Excessive HA synthesis promotes HPASMC pro-remodeling phenotype and abnormal pulmonary arterial contractility A: Quantification of extracellular HA secretion induced by Ad^CMV^-HAS2 infection of HPASMCs after 72 hours. B: Viable cell count determined by Trypan blue exclusion. The live cell count for each MOI was normalized against the equivalent MOI for the mock transduction (Ad^CMV^-GFP) control. C: Retention of exogenously supplemented HMWHA within cellular supernatants after 72 hours. D: Feedback of exogenous HMWHA on control and IPAH HPASMC proliferation after 72 hours determined by BrdU incorporation. E: Impact of Ad^CMV^-HAS2 on HPASMC proliferation (72 hours) in the presence of HYAL to abrogate HA-mediated negative feedback on cell proliferation. F: Impact of HMWHA on HPASMC proliferation induced by recombinant human platelet-derived growth factor-BB (PDGF-BB, 50 nM). G: Scratch wound closure assay to measure the impact of HMWHA and Ad^CMV^-HAS2 overexpression on PASMC migration. In these experiments, confluent cell monolayers were abraded and immersed in ultra-low serum (0.1% FBS) media with Mitomycin C (1 μM) to arrest proliferation. H, J: Measurement of HPASMC mitochondrial permeability in response to depolarization with CCCP (10 μM for 2-4 hours) using MitoCapture. Relative permeability is determined as the ratio of green (egressed from mitochondria) to red (retained in mitochondria) fluorescence. I, K: Measurement of HPASMC apoptosis by (I) caspase-3 activity, corresponding to early-to-intermediate apoptosis, determined by DEVD cleavage, and (K) Annexin-V staining, corresponding to intermediate-to-late apoptosis, determined by flow cytometry. Data expressed as mean ± SEM. *p < 0.05, **p < 0.01, and ***p<0.001 by 1-way ANOVA by treatment dose or condition (A – C, E, F), 2-way ANOVA of [cell type x HMWHA dose] (D) or [HMWHA x CCCP] (J, K), paired *t*-test (G).

Transduction of HPASMCs with Ad^CMV^-HAS2 increased HA secretion (Figure 4A), approximating the levels of HA hypersynthesis observed in IPAH cells (Figure 1B). HPASMCs transduced with lower HAS2 multiplicity (MOI 25) demonstrated greater proliferation measured by viable cell count (Figure 4B) whereas higher HAS2 multiplicity (MOI 100) was associated with inhibition of cell proliferation, suggesting feedback inhibition on proliferation by newly synthesized extracellular high molecular weight HA (HMWHA) consistent with previous reports [29]. HMWHA-induced HPASMC proliferative inhibition was further examined by treating control and IPAH cells with HMWHA. Exogenously applied HMWHA dose-dependently raised HA content in the media (Figure 4C) and decreased cell proliferation (Figure 4D). Critically, IPAH cells were less sensitive to HMWHA-induced proliferative arrest, suggesting potential escape from HMWHA inhibitory feedback. Consistent with this concept, contemporaneous treatment with hyaluronidase (+HYAL) to prevent extracellular HA accrual unmasked marked IPAH PASMC proliferation induced by HAS2 (Figure 4E). Next, we tested whether HA could potentiate growth factor-induced proliferation. Platelet-derived growth factor-BB (PDGF-BB) is a potent smooth muscle mitogen implicated in PH. HPASMCs showed hyperproliferation in response to PDGF-BB, and a further increase when treated jointly with HMWHA (Figure 4F). Since increased smooth muscle motility is an important component of vascular remodeling [35], we tested whether HA could impact cellular migration. In a scratch wound closure assay, treatment with HMWHA or overexpression of HAS2 accelerated HPASMC migration and ingress (Figure 4G).

We next examined the effects of HMWHA on HPASMC apoptosis. HPASMCs were treated with HMWHA or transduced with Ad^CMV^-HAS2, and apoptosis was induced by mitochondrial depolarization with carbonyl cyanide m-chlorophenyl hydrazone (CCCP). Mitochondrial membrane permeability was monitored using MitoCapture ratiometric fluorimetry. Both HMWHA (10 mcg/mL) and Ad^CMV^-HAS2 (MOI 50) inhibited CCCP-induced mitochondrial depolarization (Figures 4H and 4J). Treatment with HMWHA abrogated intermediate and late apoptosis, assessed by caspase-3 activity and Annexin-V fluorescence, respectively (Figures 4I and 4K). Therefore, high levels of HA promote apoptosis-resistance. HA appears to exert its anti-apoptotic action upstream in the cascade, preventing the initiation of mitochondrial depolarization. Collectively, these studies indicate that HA confers proliferative, pro-migratory, and anti-apoptotic effects on HPASMCs that are sensitive to both dose and context.

### Vessel-associated HA regulates pulmonary arterial contractility

**Figure 5.**
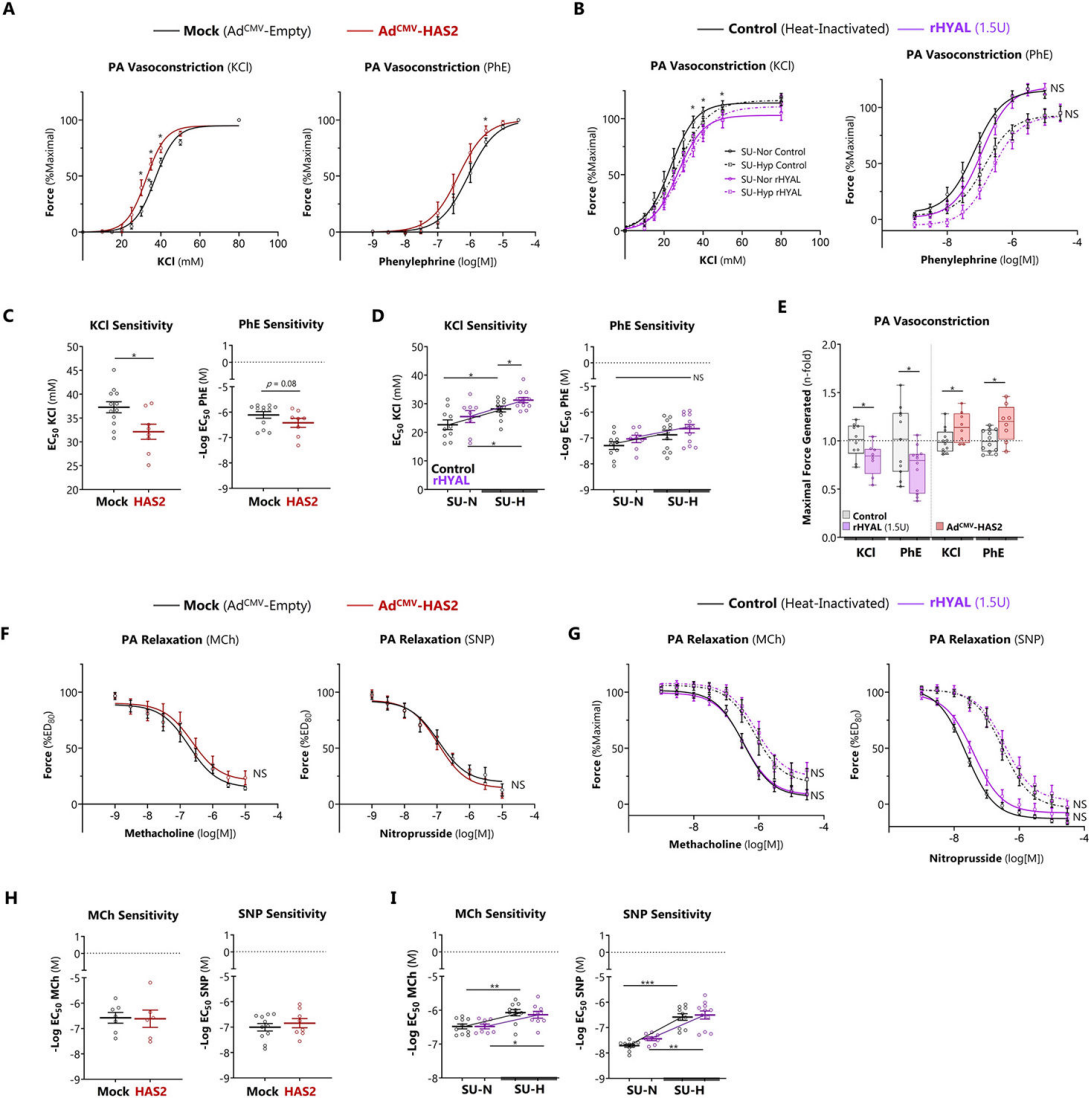
HA acutely regulates pulmonary arterial contractility. A, C: Overexpression of HA with Ad^CMV^-HAS2 heightened the dose response sensitivity to KCl, indicated by leftward shift in the response curve (A) and lower EC_50_ computed using Hill logistic fit (C, KCl panel). B, D: Acute enzymatic depletion of HA in SU-HYP arteries with rHYAL lowered dose response sensitivity to KCl, indicated by a significant rightward shift in the response curve (B) and higher EC_50_ (D, KCl panel). E: Adenoviral HA overexpression increased absolute PA contractile force generation in response to KCl and phenylephrine, whereas the opposite effect was seen with acute HA digestion. F, H: Overexpression of HA with Ad^CMV^-HAS2 had no effect on the dose response sensitivity to vasodilation with methacholine (MCh) or sodium nitroprusside (SNP), indicated by no change in the response curve (F) or EC_50_ of either agonist (H). G, I: Induction of SU-HYP PH impaired vascular relaxation responses to MCh and SNP as expected (G, dashed tracings). Acute enzymatic depletion of HA had no effect on vasodilator sensitivity, indicated by no change in the response curve (I) or EC_50_ (K) Data represented as mean or log-transformed mean (for EC_50_ values) ± SEM. *p < 0.05, **p < 0.01, and ***p < 0.001 by 2-way ANOVA of [Ad^CMV^ group x agonist dose] (A,F), [rHYAL group x agonist dose] (B,G), or [rHYAL group x rat group] (D,I), unpaired Welch’s t-test (C,E,H). Two control samples were removed from analysis because they were failed to achieve >25% contraction or relaxation and fulfilled the ROUT test as outliers.

We previously showed [5] with atomic force microscopy that excessive HMWHA increased smooth muscle cell stiffness (Young’s modulus) that was mediated by RhoA/ROCK. Therefore, we tested the impact of HA on vasomotor function of the pulmonary arteries in rodents with and without experimental pulmonary hypertension. Resistance PA segments were dissected from lungs of control (SU-NOR) or PH (SU-HYP) rats. HA was overexpressed by adenoviral transduction and depleted by treatment with recombinant hyaluronidase. Myography (Supplemental Figure S4) was performed immediately following these interventions. With regards to vasoconstriction, adenoviral overexpression of HA increased vasoconstriction to both KCI and phenylephrine (Phe, Figure 5A). Treatment with recombinant hyaluronidase (rHYAL) inhibited KCI but not Phe-induced vasoconstriction from isolated rat PAs exposed to SU-Nor or SU-Hyp (Figure 5B). Assessment of dose sensitivity to vasoconstrictor agonists showed reduced sensitivity for KCI but not Phe following HAS2 overexpression or in isolated PAs from SU-Hyp rats treated with KCI or Phe (Figure 5C and 5D). Maximal contractile force analysis demonstrated that rHYAL treatment was able to reduce KCI and Phe-induced contractility and that overexpression of HAS2 lead to augmented contractile responses (Figure 5E). With regards to vasodilation, endothelium-dependent relaxation was impaired in SU-HYP arteries as reported previously [36]. However, neither HA overexpression nor depletion had a significant impact on nonspecific or agonist-induced vasorelaxation (Figure 5F-G). However, although HAS2 overexpression did not alter MCh or SNP sensitivity, heightened sensitivity for MCh and SNP was observed in SU-Hyp PAs compared Su-Nor groups (Figure 5 H-I). Overall, these studies demonstrate that overexpression of HA led augmented contractile responses in pulmonary arteries without impacting vasodilation, and the vasoconstrictive effect was attenuated by hyaluronidase treatment.

### Smooth muscle-targeted HAS2 overexpression induces vascular remodeling and spontaneous PH

**Figure 6.**
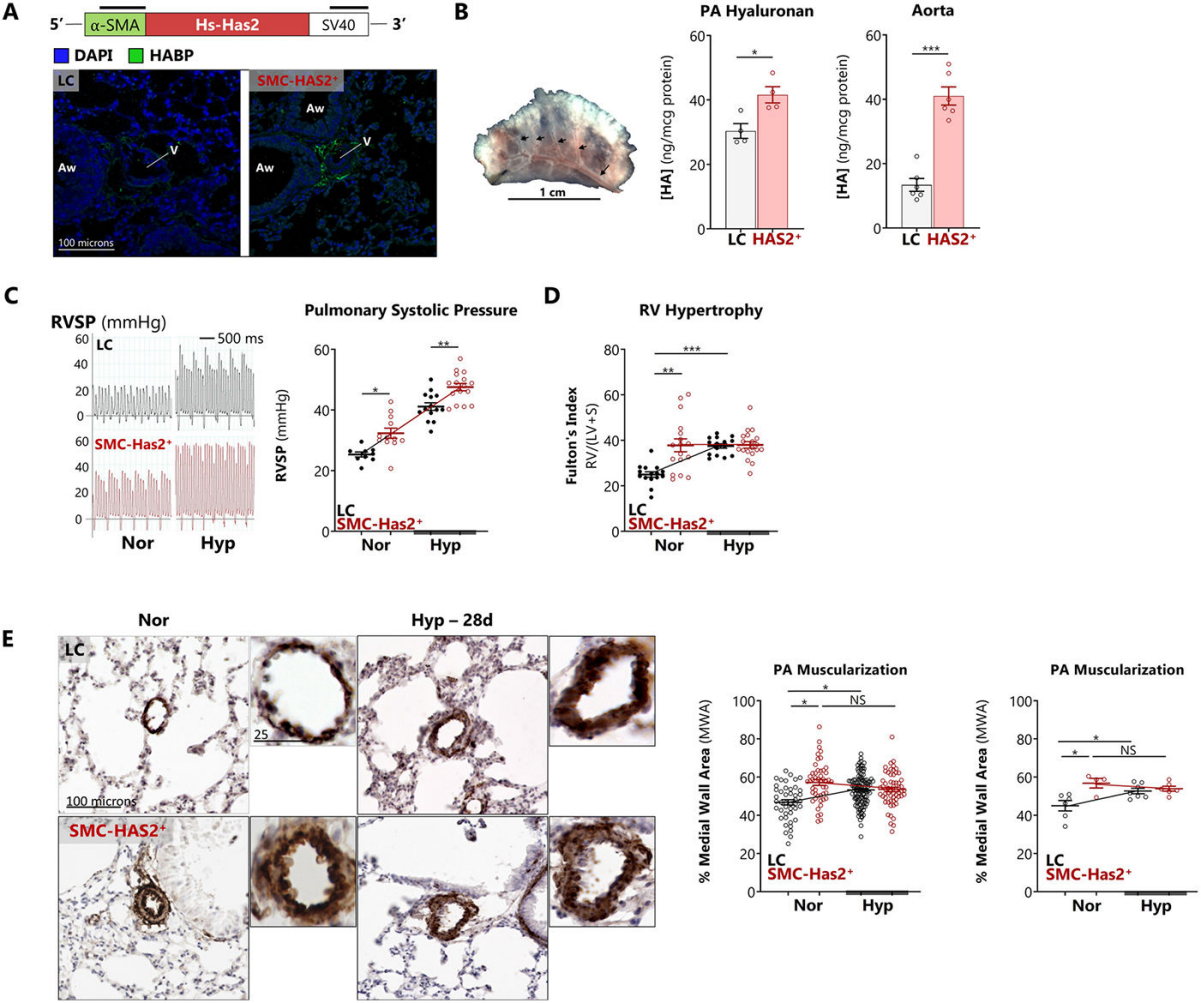
Smooth muscle-targeted HAS2 overexpression induces vascular remodeling and spontaneous PH A: Schematic of SMC-HAS2^+^ transgene structure and representative immunofluorescence images of perivascular HA in transgenic (SMC-HAS2^+^) vs littermate control (LC) mice are presented. Vascular (V) and airway (Aw) profiles are indicated. B: Representative image of PA dissection from the mouse lung. Black arrowheads: intrapulmonary PA branches. HA enrichment in isolated aortas and pulmonary arteries from SMC-HAS2^+^ mice was confirmed by ELISA. C, D: SMC-HAS2^+^ mice and their littermate controls (LC) were exposed to chronic hypoxia (10% O_2_ for 14 or 28 days) to induce PH. (C) Representative closed-chest transjugular RV pressure tracings and quantitation of RV systolic pressure (RVSP) and (D) RV hypertrophy. E: Pulmonary vascular remodeling was assessed in αSMA-stained lung sections from LC vs SMC-HAS2^+^ mice. Data are expressed as mean ± SEM. *p < 0.05, **p < 0.01, and ***p < 0.001 by Welch’s unpaired *t*-test (B) or 2-way ANOVA of [genotype x exposure] interaction (C – E). The image processing workflow to determine medial wall area is described in Supplemental Figure S8.

To directly test the impact of HAS2 hyper-synthesis on PH pathogenesis, SMC-HAS2^+^ mice and their littermate controls (LC) were exposed to hypoxia (10% O_2_ x 28 days) to induce PH. Elevated vascular HA production in these mice was confirmed by measuring HA content in aortas and pulmonary arteries (Figures 6A and 6B). Aortic HA was higher than in the corresponding PA due to the thicker vascular media in systemic arteries. Compared to littermate controls, SMC-HAS2^+^ mice displayed elevated RVSP at rest that was exacerbated following exposure to hypoxia (Figure 6C). The transgenic mice also displayed RV hypertrophy under normoxic conditions (Figure 6D) although RVH did not worsen further with hypoxia. SMC-HAS2+ mice also developed spontaneous normoxic PA muscular remodeling, as evidenced by increased αSMA+ medial wall area (Figure 6E). No differences were noted between male and female mice (data not shown).

We also examined whether the SMC-HAS^+^ mice displayed systemic circulatory abnormalities. In the transgenic mice, there was no evidence of left ventricular hypertrophic remodeling, which is a surrogate measure of chronic systemic hypertension (Supplemental Figure S5A). Furthermore, despite spontaneous and hypoxia-induced PH, these mice had no excessive polycythemia, and hematocrit was similar in both conditions (Supplemental Figure S5B). These data indicate that PH in Has2 mice is an isolated pulmonary vascular effect, rather than an expression of LV remodeling ("Group 2 PH") or increased blood impedance [37,38]. Therefore, although Has2 mice do have modestly increased aortic stiffness [39], it is unlikely to be of sufficient hemodynamic significance to induce left ventricular hypertrophy and dysfunction.

### Blocking HA synthesis induces a quiescent energy phenotype and maintains HPASMC homeostasis

**Figure 7.**
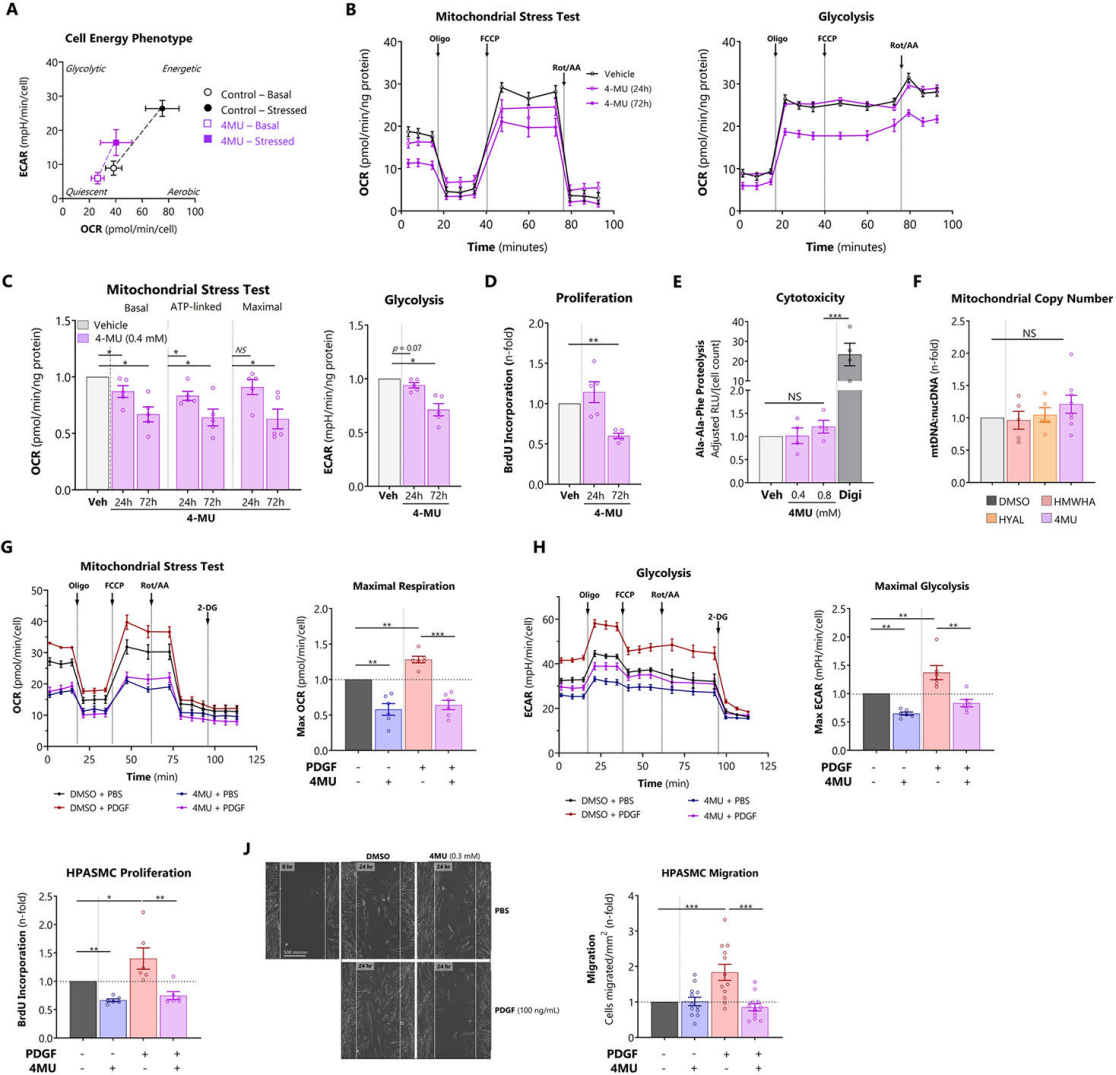
Blocking HA synthesis induces a quiescent energy phenotype and maintains HPASMC homeostasis A: Cell energy phenotype test performed in HPASMCs treated with 4MU for 24 hours. B: Representative OCR (left panel) and ECAR (right panel) profiles in HPASMCs treated for 24 or 72 hours with the HAS inhibitor 4-methylumbelliferone (4MU, 0.4 mM). C: Determination of key bioenergetic parameters. Left panel: basal, ATP-linked (O_2_ equivalents), and maximal respiration were derived from the OCR profiles. Right panel: basal and maximal glycolysis were obtained from the ECAR profiles. D: Proliferation of 4MU-treated cells measured by BrdU incorporation E: Verification that 4MU does not induce cytotoxicity at relevant doses using the MultiTox cell death assay to measure extracellular Ala-Ala-Phe cleavage. Digitonin (Dig, 2%) was used as a positive control for toxicity. F: Measurement of mitochondrial copy number by PCR for relative abundance of mitochondrial versus nuclear genomic DNA. G, H: Representative (G) OCR and (H) ECAR profiles of cells treated with the potent PH-implicated mitogen PDGF-BB (100 ng/mL) and 4MU along with determination of maximal respiration and glycolysis. I: Proliferation of HPASMCs treated with PDGF-BB (50 nM) and 4MU, assessed by BrdU incorporation. J: Scratch wound migration assay of HPASMCs treated with PDGF-BB (100 nM) and 4MU (0.3 mM). Cells were maintained in ultra-low serum (0.5%) and Mitomycin C (1 μM) to suppress proliferation. Data expressed as mean ± 95% CI (A) or mean ± SEM (B – J). *p < 0.05, **p < 0.01, and ***p < 0.001 by 1-way ANOVA by single treatment condition (dose, time, or type) (C – F), or 2-way ANOVA of [PDGF x 4MU] interaction (G-J). In all bioenergetic experiments, OCR and ECAR are normalized to total protein recovered per well or the final cell number. Additional bioenergetic indices are presented in Supplemental Figure S2, and bioenergetic index definitions are summarized in Supplemental Figure S7.

Having established that excessive HA induces mitochondrial dysfunction and a glycolytic shift in HPASMC (Figure 3), we tested whether blocking HA synthesis could restore bioenergetic function. Seahorse XF time course studies were performed on HPASMC treated with 4-methylumbelliferon (4MU), a potent HAS inhibitor. Treatment with 4MU induced a quiescent cell energy phenotype characterized by lower baseline and stressed OCR and ECAR, diminishing the total metabolic potential by 59% (Figure 7A). Subsequent mitochondrial stress testing revealed that 4MU suppressed several indices of oxidative respiration by 24 hours (Figures 7B and 7C) despite unchanged cellular proliferation at this time point (Figure 7D). At 72 hours, mitochondrial respiration and proliferation were commensurately reduced. 4MU also produced a significant antiglycolytic effect at 72 hours (Figures 7B and 7C). The loss of oxidative and glycolytic metabolism were not due to 4MU-induced cytotoxicity, assayed by extracellular proteasome peptidase activity (Figure 7E). Overall, these data indicate that the mitochondrial response to 4MU is a primary drug effect on mitochondrial function, rather than a secondary reflection of decreased cell proliferation or drug toxicity. Finally, manipulation of HA content with exogenous HMWHA, hyaluronidase, or 4MU had no impact on mitochondrial copy number, assessed by PCR measurement of mitochondrial-to-nuclear genome ratio (Figure 7F). Therefore, the bioenergetic effects of HA are likely mediated by altered mitochondrial function rather than abundance.

Next, we examined whether 4MU could normalize the glycolytic shift, the cardinal metabolic derangement of PH. We measured OCR and ECAR in HPASMCs treated with PDGF-BB and 4MU. PDGF is a powerful smooth muscle mitogen that plays a key role in pulmonary vascular remodeling [40]. PDGF increased both OCR and ECAR equally, and 4MU abrogated these effects completely (Figures 7G and 7H) consistent with induced quiescence. To link these HA-dependent bioenergetic changes to pro-remodeling HPASMC phenotypes, we queried whether 4MU could inhibit PDGF-induced cell proliferation and migration. 4MU reduced PDGF-induced proliferation (Figure 7I), analogous to the glycolytic inhibitor 3PO (Supplemental Figure S2L, including comprehensive bioenergetic data), and also prevented PDGF-induced HPASMC migration (Figure 7J).

Collectively, these results indicate that the HA inhibitor 4MU can simultaneously restore metabolic and phenotypic homeostasis to smooth muscle cells.

### PH-derived HPASMCs are highly susceptible to pharmacologic depletion of HA

**Figure 8.**
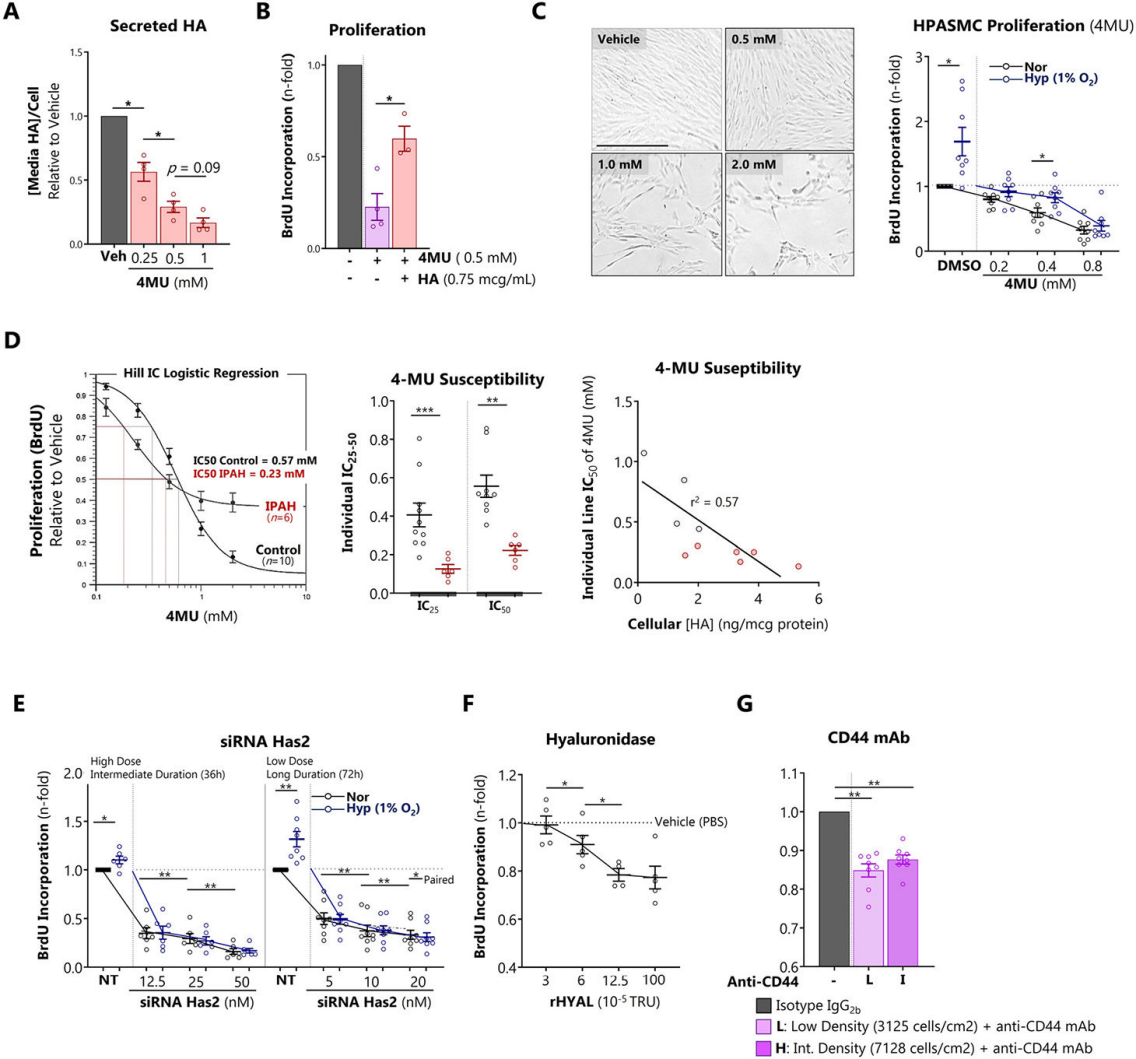
PH-derived HPASMCs are highly susceptible to pharmacologic depletion of HA A: The HAS inhibitor 4MU dose-dependently reduces HA production by HPASMCs after 72 hours. B: Partial rescue of 4MU-induced proliferative arrest by treatment with exogenous HMWHA. C: Representative widefield microscopy of HPASMCs treated with 4MU. Dose-dependent effect of 4MU on normoxic and hypoxic (1% O_2_ x 72 hours) HPASMC proliferation assessed by BrdU incorporation. D: Effect of 4MU on the proliferation of control versus IPAH PASMCs. E: siHAS2 induces dose-dependent proliferative inhibition of normoxic and hypoxic HPASMC at 36 and 72 hours. F: HA lysis with HYAL induces dose-dependent HPASMC proliferative inhibition after 48 hours. G: Neutralization of CD44 inhibits HPASMCs at low and intermediate densities. Data expressed as mean ± SEM. *p < 0.05, **p < 0.01, and ***p < 0.001 by 1-way ANOVA of treatment condition (A, B,G) or time point (F), 2-way ANOVA of [oxygen group x 4MU group] (C) or [oxygen group x siRNA group] (E), and Welch’s unpaired *t*-test (D).

To further probe the impact of HA on proliferation, we used several strategies to deplete HA in HPASMCs. Treatment with 4MU dose-dependently blocked HPASMC HA production (Figure 8A) and inhibited proliferation, an effect partially reversed by replenishing extracellular HA (Figure 8B). 4MU also reduced increases in HPASMC proliferation *in vitro* under hypoxic (1% O_2_) conditions (Figure 8C). However, hypoxia caused chemoresistance to 4MU, evidenced by a 27.8% reduction in the antiproliferative potency of 4MU across all doses. This was attributable to hypoxic alterations in 4MU metabolism, regulated by competition between glucuronidation and glycolysis (Supplemental Figure S6). Compared to control HPASMCs, IPAH PASMCs demonstrated enhanced sensitivity to HA blockade with 4MU, more efficiently inhibiting their proliferation (Figure 8D, left and middle panels). The degree of inhibition by 4MU was also inversely correlated with the basal level of HA produced by each cell line (Figure 8D, right panel) corroborating the HA-dependency of basal proliferation. Since HAS2 is the predominant synthase for HMWHA in the lung, we hypothesized that HAS2 siRNA would exert an antiproliferative effect on HPASMCs. HAS2 siRNA dose-dependently attenuated cell proliferation at 36 and 72 hours with greater antiproliferative efficiency in hypoxia (Figure 8E). Digestion of HA with hyaluronidase also attenuated proliferation (Figure 8F), consistent with previous reports that the pericellular HA coat is required for smooth muscle mitosis [31]. CD44 is the canonical cell surface HA adhesion molecule involved in density-dependent contact inhibition [41]. To test the requirement for CD44-HA interaction for HPASMC proliferation, we blocked CD44 with a monoclonal antibody. Neutralization of CD44 decreased cell proliferation at both low and intermediate cell densities (Figure 8G). Collectively these findings indicate that HA is required to support basal and hypoxia-exacerbated proliferation of HPASMCs.

### Blocking HA synthesis protects against PH and reverses established disease

**Figure 9.**
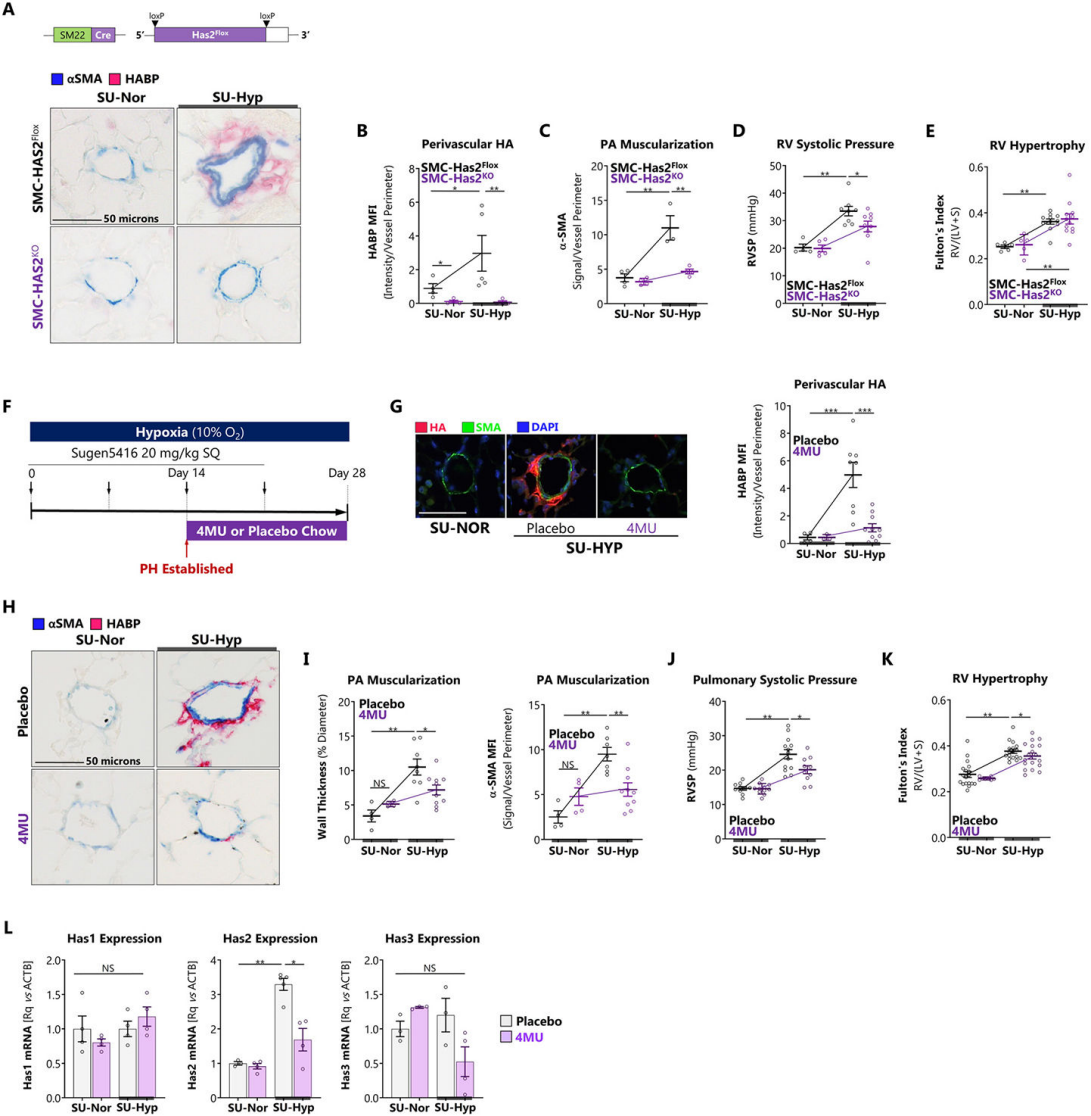
Blocking HA synthesis protects against PH and reverses established disease. A: SMC-HAS2^Flox^ and SMC-HAS2^KO^ mice were treated ± SU-HYP to induce PH. Schematic of SMC-targeted HAS2 knockout strategy and representative immunofluorescence staining for HA. B: Immunofluorescence quantitation of pulmonary vessel-associated HA in the SMC-HAS2 knockout mice verifying absence of perivascular HA. Scale bar = 50 microns. C – E: (C) Assessment of vessel muscularization with α-SMA staining. (D) Measurement of RVSP and (E) RV hypertrophy. F: Schematic of the experimental model for treating C57Bl/6 mice with the HAS inhibitor, 4MU. Mice received weekly subcutaneous injections of Sugen5416 during exposure to hypoxic (10% O_2_) or normoxic conditions. In this model, RVSP demonstrated onset of PH by 14 days. Mice were treated with 4MU (125 mg/kg) versus placebo chow starting day 14 and ending at day 28. G: Immunofluorescence staining and quantitation of pulmonary vessel-associated HA in the 4MU-treated mice. Scale bar = 50 microns. H, I: (H) Representative histology and (I) quantification of pulmonary vascular muscular remodeling by vascular wall thickness and fluorescent signal intensity (MFI) in mice with PH treated with 4MU. Scale bar = 50 microns. J, K: Measurement of (J) RVSP and (K) RV hypertrophy in mice treated with 4MU. L: Whole lung expression of HAS isoforms (Rq: relative expression as fold-change over control; ACTB: beta actin). Data expressed as mean ± SEM. *p < 0.05, **p < 0.01, and ***p<0.001 by 2-way ANOVA of [genotype x exposure] (B – E), [4MU group x exposure] (G – L).

Taken together our results point at a pathophysiological effect of elevated HAS2 expression as a driver of metabolic dysfunction, proliferation and migration that contribute to vascular remodeling. Thus, to test whether targeted inhibition of HAS2 protects against PH, we generated mice with conditional deletion of HAS2 in smooth muscle cells (SMC-HAS2^KO^) and induced PH with exposure to SU-HYP. SMC-specific deletion of HAS2 eliminated induction of pulmonary vascular HA under PH-inducing conditions supporting the critical role of this cellular compartment and HAS isotype in HA production (Figure 9A). SMC-HAS2^KO^ attenuated pulmonary vascular HA and α-SMA deposition following SU-HYP (Figures 9B and 9C). SMC-HAS2^KO^ mice were protected against SU-HYP-induced increases in RVSP (Figure 9D) but not RVH (Figure 9E). To further test the ability of HAS blockade to attenuate PH, SU-HYP-exposed mice were treated with 4-methylumbelliferone (4MU), a potent pan-HAS inhibitor according to the experimental schema shown in Figure 9F. 4MU inhibits HA by depleting the cytoplasmic pool of UDP-glucuronic acid and via transcriptional repression of HAS2. Initiation of 4MU at day 15, once vascular remodeling was present (Figure 1H), prevented the deposition of perivascular HA (Figure 9G), decreased pulmonary vascular muscularization (Figure 9H and 9I), and attenuated SU-HYP-induced elevation of RVSP (Figure 9J) and RVH (Figure 9K). 4MU decreased *has2* expression in lung tissue without altering the expression of *has1* or *has3* (Figure 9L), further highlighting the specific relevance of this isoform in PH pathogenesis. These studies indicate that genetic deletion or pharmacologic inhibition of HA can protect against experimental PH and support anti-HA therapy as a viable therapeutic strategy.

## DISCUSSION

This study provides new evidence that hyaluronan (HA) directly contributes to the pathogenesis of PH. Our main findings can be summarized in three central themes: (1) a novel mechanism for dysregulation of the HA-HAS2 axis in PH involving depletion of NUDT21 and subsequent *has2* 3’UTR shortening; (2) the pro-remodeling effect and cellular bioenergetic dysfunction induced by excessive HA in the pulmonary vasculature; and (3) the therapeutic promise of HA blockade in additional preclinical models of PH. In concert with evolving literature, the new findings of our study support the following sequence of events: hypoxic conditions trigger loss of NUDT21, promoting *has2* 3’UTR shortening with subsequently increased *has2* expression, stimulating progressive accumulation of HA that initiates and contributes to vascular remodeling in PH.

### Hyper-synthesis of HAS2 and HA accumulation in PH

Consistent with previous reports [4-6,8,42], we observed pathologic elevation of HA in human lungs and across several rodent models of PH. Our findings add to previous results by establishing pulmonary arterial smooth muscle as a predominant site of HA dysregulation and by identifying HAS2 as the dysregulated synthase isoform. Histological examination of rodent and human PH specimens revealed pulmonary vascular deposition of HA, which steadily increased during the evolution of PH. In SMC-HAS2^+^ mice, simultaneous RNA *in situ* hybridization and HA staining demonstrated medial expression of HAS2 but *adventitial* accumulation of the HA product [39]. This implies that HA synthesized in the smooth muscle cell layer is extruded outwards, consistent with the intimal-basolateral gradient observed for heparan sulfate, another prominent vessel glycosaminoglycan [43]. It is also possible that HAS2 expression is upregulated in cells closer to the adventitia contributing to higher perivascular-HA deposition.

Levels of HA are determined by the local balance of anabolic (synthase: HAS) and catabolic (hyaluronidase: HYAL) processes. Accumulation of HA was previously attributed to low plasma hyaluronidase activity observed in human patients with PH [4]. However, whether these results reflected causation or association was not known. Indeed, both direct and reverse zymography of lung tissues showed elevated lung tissue hyaluronidase activity during the evolution of monocrotaline-induced PH in rats [28]. Our study offers an additional perspective, demonstrating that increased HAS activity is present in pulmonary artery smooth muscle cells (PASMCs), where it is both necessary and sufficient to induce spontaneous PH.

The significance of the smooth muscle-derived HA is further evidenced by elevation of HA secreted from PASMCs isolated from IPAH patients, persisting even after serial passage. These findings suggest a permanent change in the HA biosynthesis machinery related in part to aberrant post-transcriptional processing of *has2.* HAS isoforms are known to be dynamically regulated at the transcriptional, post-transcriptional and post-translational levels [44]. Yet despite the cloning of the HAS isoforms over 30 years ago, the pathological mechanisms that lead to increased expression in disease are not fully understood A recent review on the transcriptional and translational control HAS2 activity identified several mechanisms that control its expression. In contrast with HAS1 and HAS3, the HAS2 gene has one transcript and an antisense RNA (HAS2-AS1) [45]. In this study, we determined that the pre-mRNA polyadenylation and cleavage regulator NUDT21 is downregulated in PAs isolated from explanted lungs from patients with PAH and in SU-HYP exposed mice. We demonstrate, using *in vitro* approaches that loss of NUDT21 leads to shortening of the *has2* 3’UTR, resulting in HAS2 upregulation and HA hyper-synthesis. 3’UTR shortening results in loss of regulatory sites, allowing transcripts such as *has2* to escape sponging and decay, leading to increased translation [20]. This finding concurs with recent reports demonstrating that loss of NUDT21 leads to increased profibrotic gene transcription in the skin and lungs [20,46]. NUDT21 deficiency and consequent 3’UTR shortening has been shown to drive neoplastic processes [22-24,47] through de-repression of oncogenes. Therefore, it is not unexpected that NUDT21 depletion plays a central role in PH, a condition marked by fibrotic vascular stiffening and cancer-like hyperplasia of pulmonary arterial wall cells.

Upstream mechanisms of NUDT21 depletion require further investigation. Systemic hypoxemia is prevalent in PH [25], and inappropriate normoxic stabilization of HIF1α and HIF2α contributes to PH pathogenesis [48]. NUDT21 has recently been confirmed as a HIF1α responsive target, accounting for hypoxia-induced loss of NUDT21 in non-small cell lung cancer cells [49]. HIF1α is well-understood to be a transcriptional activator, but it also functions in rare instances as a direct repressor. Further studies are needed to clarify the direct and indirect regulation of NUDT21 by hypoxia inducible factors. Independent of hypoxia signaling status, unbalanced TGFβ family activation is a pervasive finding in experimental and human PH [50] that is being targeted in ongoing clinical trials [51]. Increased TGFβ signaling induces miR-203 to target the 3’ UTR of NUDT21 [19] accounting for its repression. The interplay of hypoxia, TGFβ, and NUDT21-related changes in hyaluronan and other ECM components therefore appears to be a highly relevant network in PH.

### HAS2 hyper-synthesis promotes vascular remodeling and bioenergetic dysregulation

Having established that HA is upregulated in PH, we next studied the impact of HA on the pulmonary vasculature. We found that HA overload induces a proliferative and apoptosis-resistant PASMC phenotype that promotes vascular remodeling and PH *in vivo.* Regulation of vascular smooth muscle proliferation by HA involves a balance between the rate of intrinsic HA synthesis and feedback inhibition by newly formed extracellular HA. As the cells proliferate, expansion of a HA-rich pericellular coat serves as a transitional matrix required for replication. Therefore, HMWHA supports basal vascular smooth muscle cell proliferation; once formed, however, HA appears to exert negative feedback on PASMC proliferation [29]. Growth suppressive mechanisms of HMWHA are known to involve CD44-dependent Hippo pathway signaling as well as engagement of early contact inhibition through Ezrin-Radixin-Moesin (ERM) actin-binding proteins [41]. Given that high levels of extracellular HMWHA oppose proliferation *in vitro,* our observation that HAS2 overexpression increases vascular remodeling *in vivo* is quite surprising. It is possible that excessive HA facilitates mitogen-induced proliferation, since vasoactive growth factors such as TGFβ, PDGF, serotonin, and endothelin-1 are locally and systemically elevated in PH. Alternatively, it is conceivable that HAS2 overexpression escapes negative feedback, perhaps by inducing cell-autonomous changes in gene expression regulating proliferation. Finally, excessive deposition of HMWHA may also furnish a substrate for cleavage into lower molecular weight fragments, which participate in paracrine signaling in a variety of cell types relevant to vascular remodeling.

While PASMC hyperproliferation may dominate early in the disease course, advanced PH involves a shift towards apoptosis-resistance [52]. HMWHA has been reported to serve a cytoprotective role against the presence of intrinsic and external cell death signals ranging from mitochondrial reactive oxygen species to inflammatory cytokine exposure [53-55]. We show that HA accumulation contributes to PASMC apoptosis-resistance suggesting that the copious matrix in which PH vessels are embedded could sustain the apoptosis-resistant phenotype of the vascular cells and serve as a therapeutic target for reversing pulmonary vascular remodeling in PH.

In addition to its pro-remodeling effects, we also observed that HA positively regulates pulmonary artery contractile force production. This is consistent with previous studies from our group showing increased cell stiffness following HA treatment that was RhoA/ROCK-dependent [5]. Traditionally, vascular smooth muscle cells are thought to undergo phenotypic switching between a contractile stationary state and a hyperproliferative migratory synthetic state. As expected, our studies showed that induction of SU-HYP PH in rats blunted pulmonary artery contractility relative to control animals, consistent with classical phenotypic switching [36,56]. Despite promoting a hyperproliferative and hypermigratory phenotype in PAMSCs, overexpression of HA also stimulated vessel contractility. The population of smooth muscle cells in the vessel wall is likely constant; therefore, the enhanced whole-vessel contractility may be explained by increased contractility per myocyte or by formation of an HA-rich perivascular matrix that facilitates vessel constriction. This latter explanation is supported by gel contraction studies in which incorporation of HA into the gel matrix enhanced tangential collagen organization and tethering in smooth myocytes within a similarly acute timeframe (18 hours) in a CD44-dependent manner. From these cell and vessel studies, we can infer that chronic upregulation of intrinsic HA synthesis in PASMC promotes vascular smooth muscle pro-remodeling phenotypic switching, whereas extrinsic HA modulates pulmonary vasomotor tone. Excessive HA could therefore resolve the seemingly discordant observations of inappropriate vasoconstriction in spite of hyperproliferation in PH [57].

The metabolic theory of PH [58,59] postulates that changes in cellular metabolism dictate pathological remodeling phenotypes such as proliferation, apoptosis-resistance, and inflammation. As a corollary, correction of metabolic derangements may restore homeostasis in pulmonary vascular cells [60-62]. The ECM has emerged as a key regulator of metabolism; cells react to their matrix, responding to local biophysical cues such as stiffness and topography, to orient their metabolic programs [3,17,18]. Because it is a major constituent of the perivascular ECM, we tested whether excessive HA contributes to altered metabolism in PASMC. Our extracellular flux analysis revealed that high levels of HA reprogrammed PASMC towards a glycolytic cell energy phenotype coupled with a decrease in ATP-linked mitochondrial respiration, recapitulating the Warburg effect that is characteristic of vascular wall cells in PH. These findings are akin to a recent report wherein HA also suppressed mitochondrial oxidative respiration and sustained a glycolytic proliferative state in mesenchymal stem cells [63]. Furthermore, pharmacologic depletion of HA with 4MU prevented the glycolytic shift induced by PDGF-BB. These findings complement other studies in non-vascular cells indicating that HA directly impacts cellular energy homeostasis [64,65].

### The therapeutic promise of HA blockade

In this study, we investigated the approach of blocking HA synthesis using an oral small molecule inhibitor, 4MU, also known as hymecromone. We showed that 4MU could reverse established PH and attenuate pulmonary vascular remodeling in several murine models designed to simulate primary pulmonary arterial hypertension (SU-HYP model) as well as PH associated with chronic lung disease such as pulmonary fibrosis and emphysema [5,42]. We confirmed that 4MU targets the hypersynthetic HA-overloaded phenotype of diseased smooth muscle cells, preventing their proliferation and migration. These results are consistent with previous findings that 4MU reduces the migration of aortic SMCs through a CD44/HA-dependent mechanism [66]. The beneficial role of 4MU has also been documented in cancer cell lines where it abrogated cancer cell proliferation and migration through inhibition of HAS2 [67]. In line with our studies showing that inhibition of HAS2 abrogates hypoxia-induced vascular remodeling, inhibition of angiogenesis following HAS2 endothelial degradation has been reported [68].

4MU has an excellent safety profile and is clinically approved in Italy to treat biliary dyskinesia and benign prostatic hypertrophy. The published clinical experience with 4MU now spans 216 individuals, including 143 healthy volunteers [69,70]. Recently, orally administered 4MU was shown to dose-dependently reduce sputum HA levels with no significant adverse events, demonstrating its favorable therapeutic profile to target pulmonary pathology [71]. Although 4MU was well-tolerated, a significant limitation is its low oral bioavailability of 3%. Hepatic glucuronidation converts the 4MU prodrug to 4MUG for excretion in the bile. This poses a unique pharmacologic dilemma since 4MUG is also its active metabolite [72] by virtue of its competitive action at the glucuronic acid binding site of HAS2. Ongoing development of 4MUG and related ester derivatives may overcome this challenge [73]. In parallel with reduction of PH *in* vivo, we also confirmed that 4MU decreased the glycolytic shift in proliferating PASMCs. Our data that 3PO overcomes hypoxic resistance to 4MU, paired with existing literature showing synergy between 4MU and dichloroacetate, imply that the efficacy of 4MU may be further enhanced if paired with a glycolytic inhibitor.

Clearance of excessive HA can be accomplished by augmenting HYAL or inhibiting HAS. Selection of the appropriate strategy is a key biological distinction with clinical importance. For example, recombinant HYAL therapy is approved to facilitate systemic absorption of subcutaneous drugs [74]. However, HYAL treatment may lead to uncontrolled HA depolymerization to generate low molecular weight (LMHWA) fragments, which may impart deleterious pro-inflammatory signaling effects [75,76] contributing to pulmonary vascular pathology [29]. When given systemically at doses intended to eliminate HA from visceral tissues, mice injected with recombinant HYAL display signs of systemic inflammation such as torpor, anorexia, and weight loss. From the standpoint of effect size, depletion of HA by blocking *de novo* HA synthesis exerted greater antiproliferative activity (4MU: 40.5%, siHAS2: 62.6%) compared to lysis or interference with preassembled extracellular HA (HYAL: 21.6%; anti-CD44 mAb 15.1%), lending further credence to the concept that HAS inhibition may be more potent than HYAL augmentation. Finally, inhibition of *de novo* HA production offers the ability to monitor and adjust treatment. Plasma levels of HA are elevated in PH and correlate tightly with disease severity [7], offering a mechanistic biomarker for a personalized medicine approach to titrate anti-HA therapy with 4MU. In contrast, injection of HYAL produces a rapid and prominent rise in plasma HA [77], likely reflecting release of HA from the vascular glycocalyx and shedding into the systemic circulation. These concerns, paired with greater cost and need for parenteral delivery, may be significant detractors from HYAL-based HA reduction therapy.

Limitations of the present work include the lack of inducible HA expression or knockout *in vivo.* Since HA is likely vital for normal lung morphogenesis [78], congenital loss or overexpression of vascular HAS2 may introduce lesions in lung development. Since HAS2 is markedly induced in a time- and severity-dependent fashion in disease, future studies should consider the use of temporally controllable conditional HA-HAS2 models. In this study, our analysis was restricted to the effect of HA specifically in pulmonary arterial smooth muscle. The pathobiology of PH involves a complex heterocellular environment consisting of endothelium, smooth muscle, adventitial fibroblasts, tissue macrophages, and circulating immune cells [79]. Further work is required to delineate the impact of excessive HA on these other critical cell types and their contribution to HA homeostasis in the distal pulmonary vascular unit. The present study focused on the anabolic arm of HA turnover, identifying excessive HMWHA as a driver of PH. On the catabolic side, the action of low molecular weight (LMWHA) fragments on vascular inflammation and remodeling are likely important as well inviting future study of the concerted regulation of HA synthases, hyaluronidases, and HA fragments in the context of inflammatory pulmonary vascular remodeling. Lastly, it is recognized that proliferative remodeling in PH occurs most actively in the terminal pre-arteriolar vessels and that myocytes from the proximal PA branches have a different proliferative capacity than those isolated from the distal vasculature [52,80]. This pulmonary vascular remodeling involves not only proliferation and apoptosis-resistance, but mobilization, transdifferentiation, and migration of smooth muscle down the longitudinal axis of the pulmonary arterial tree . Future studies interrogating HA regulation of these morphodynamic features of smooth muscle remodeling will be required to appreciate this complex pathobiology more completely.

Strengths of our study include the inclusion of HPASMCs from multiple patients with IPAH to better capture the biological variability present in the clinical population. To enhance the internal validity of our findings, we demonstrate the pathogenic role of excessive HA using genetic and pharmacologic gain- and loss-of-function in multiple models of PH *in vivo.* Finally, we established the ability of 4MU, a small molecule HAS inhibitor with a well-established clinical safety profile, to reverse pro-remodeling phenotypes in smooth muscle cells and preexisting PH in mice.

## Supplementary Material

Supplementary materials 1

Supplementary materials 2

## Nonstandard Abbreviations and Acronyms:

3PO: 3-(3-Pyridinyl)-1-(4-pyridinyl)-2-propen-1-one
3’UTR: 3’ untranslated region
4MU: 4-methylumbelliferone (hyaluronan synthase inhibitor)
APA: alternative polyadenylation
BrdU: 5-bromo-2′-deoxyuridine
CCCP: carbonyl cyanide *m*-chlorophenyl hydrazone
CFIm: cleavage factor 1m, mammalian
dPAS: distal poly-(A) signal
ECAR: extracellular acidification rate (index of glycolysis)
ECM: extracellular matrix
FCCP: carbonyl cyanide-4-(trifluoromethyoxy)phenylhydrazone
HA: hyaluronan
HABP: HA binding protein
HMWHA: high molecular weight HA (> 1000 kilodaltons)
HAS: HA synthase
HYAL: hyaluronidase
HYP: hypoxia
HPASMCs: human pulmonary artery smooth muscle cells
IP: intraperitoneal
IPAH: idiopathic pulmonary arterial hypertension
KO: knockout
MOI: multiplicity of infection
NOR: normoxia
NUDT21: nudix hydrolase 21
Oligo: oligomycin
OCR: oxygen consumption rate (index of mitochondrial respiration)
PA: pulmonary artery
PDGF-BB: platelet-derived growth factor-BB
PH: pulmonary hypertension
RT-qPCR: quantitative real-time PCR
R/A: rotenone/antimycin A
RV: right ventricle
RVSP: RV systolic pressure
RV/(LV+S): right ventricle: left ventricle + septum weight ratio (an index of RVH
α-SMA: alpha smooth muscle actin
SMC: smooth muscle cell
SMC-HAS2^+^: SMC-targeted HAS2 transgene overexpressing mouse
SMC-HAS2^KO^: SMC-targeted HAS2 deletion mouse
siRNA: small interfering RNA
SU: Sugen5416 (vascular endothelial growth factor receptor antagonist)
SU-HYP: SU combined with hypoxia (10% O_2_)
