# Supplementary material for: 3’UTR shortening of HAS2 promotes hyaluronan hyper-synthesis and bioenergetic dysfunction in pulmonary hypertension
Source: Matrix Biol. Author manuscript; Available in PMC 2023 Aug 1. (PMC9676077; doi:10.1016/j.matbio.2022.06.001)
Supplement: Supplementary materials 1 [file NIHMS1849092-supplement-Supplementary_materials_1.docx]

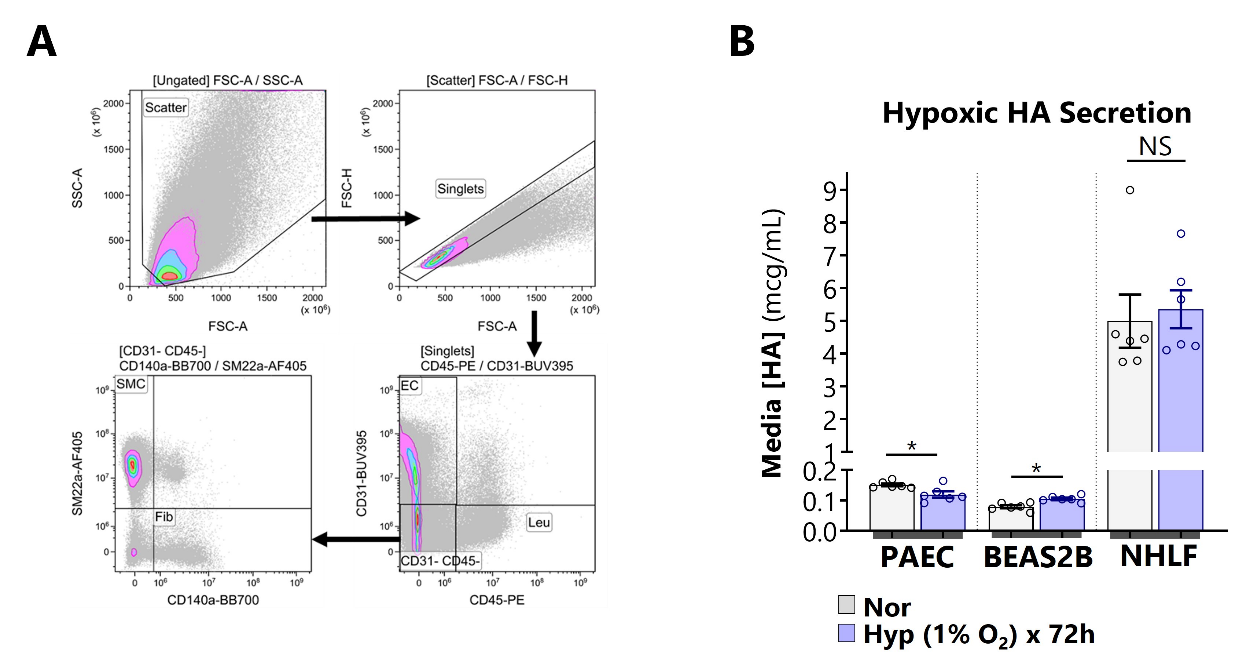


**SUPPLEMENTAL FIGURE S1**

**
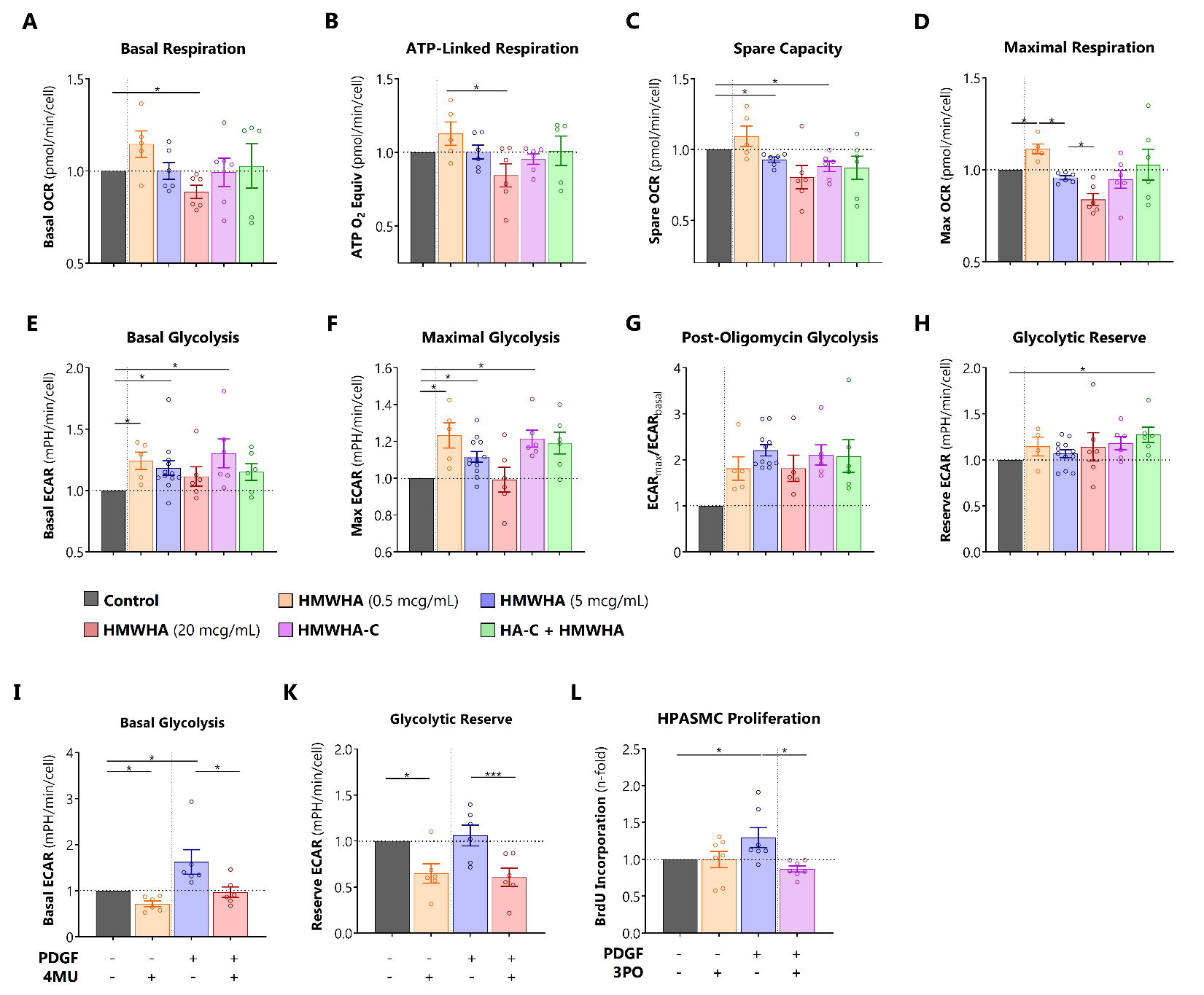
**

**SUPPLEMENTAL FIGURE S2**

**
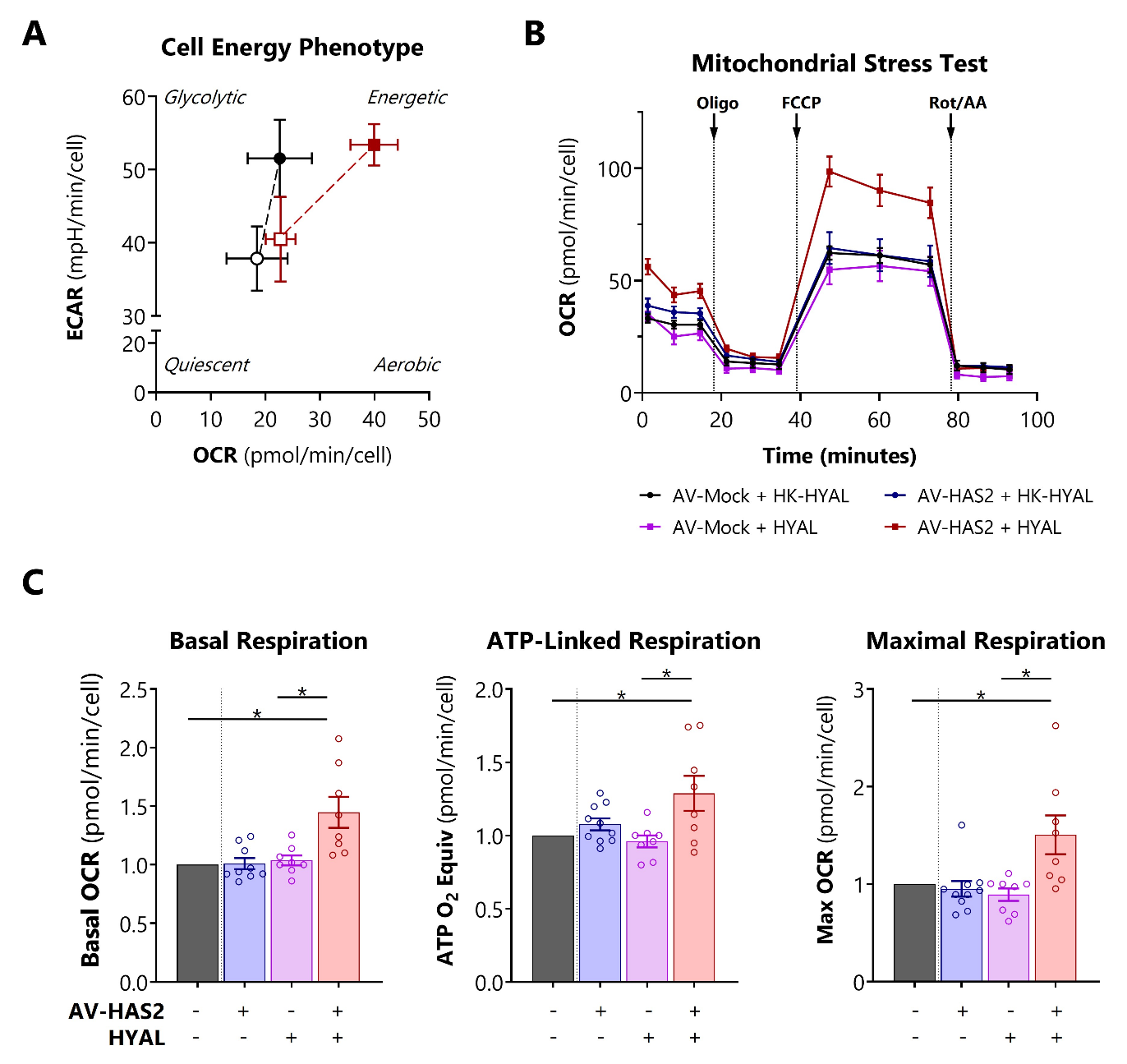
**

**SUPPLEMENTAL FIGURE S3**

**
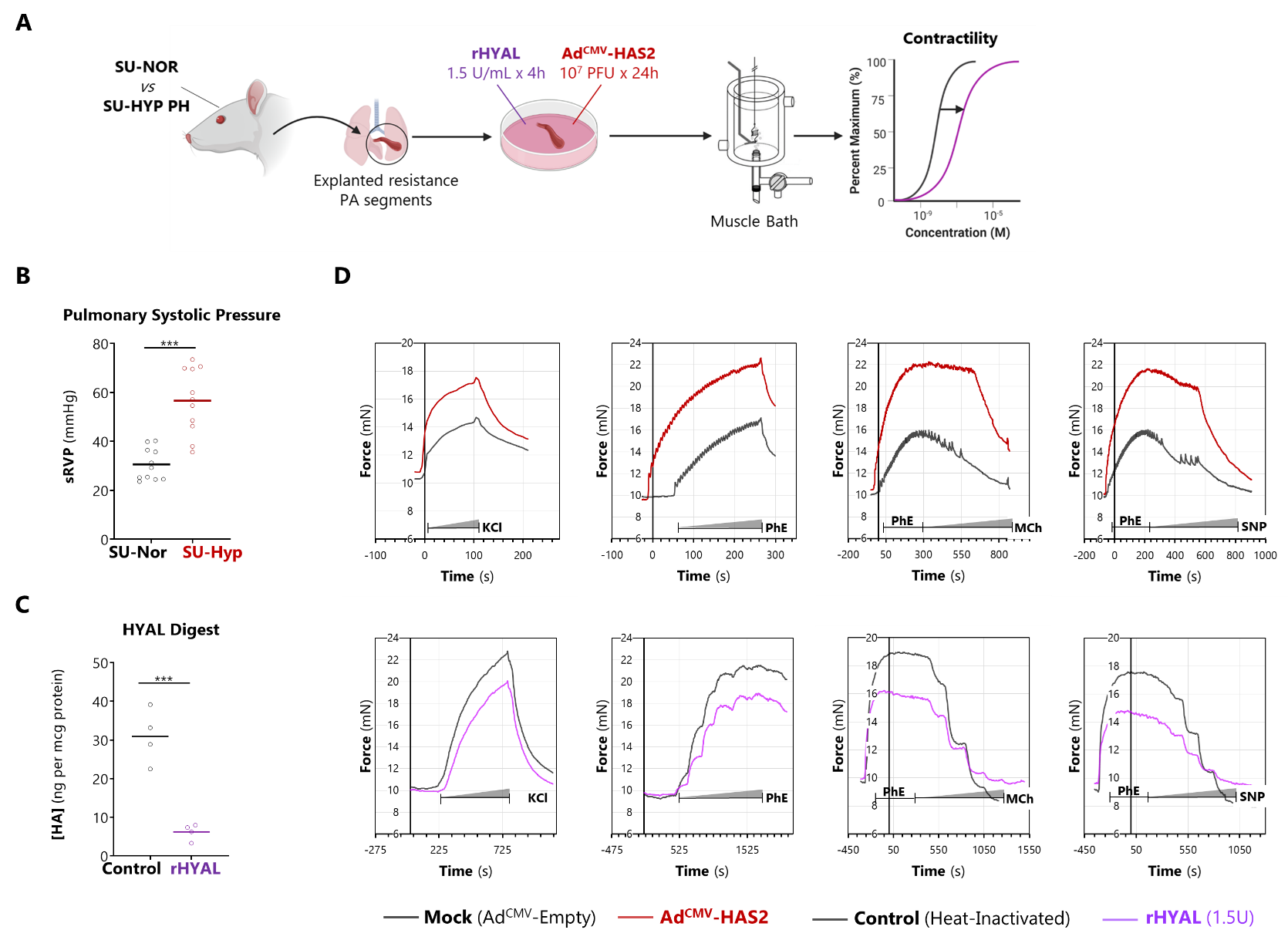
**

**SUPPLEMENTAL FIGURE S4**

**
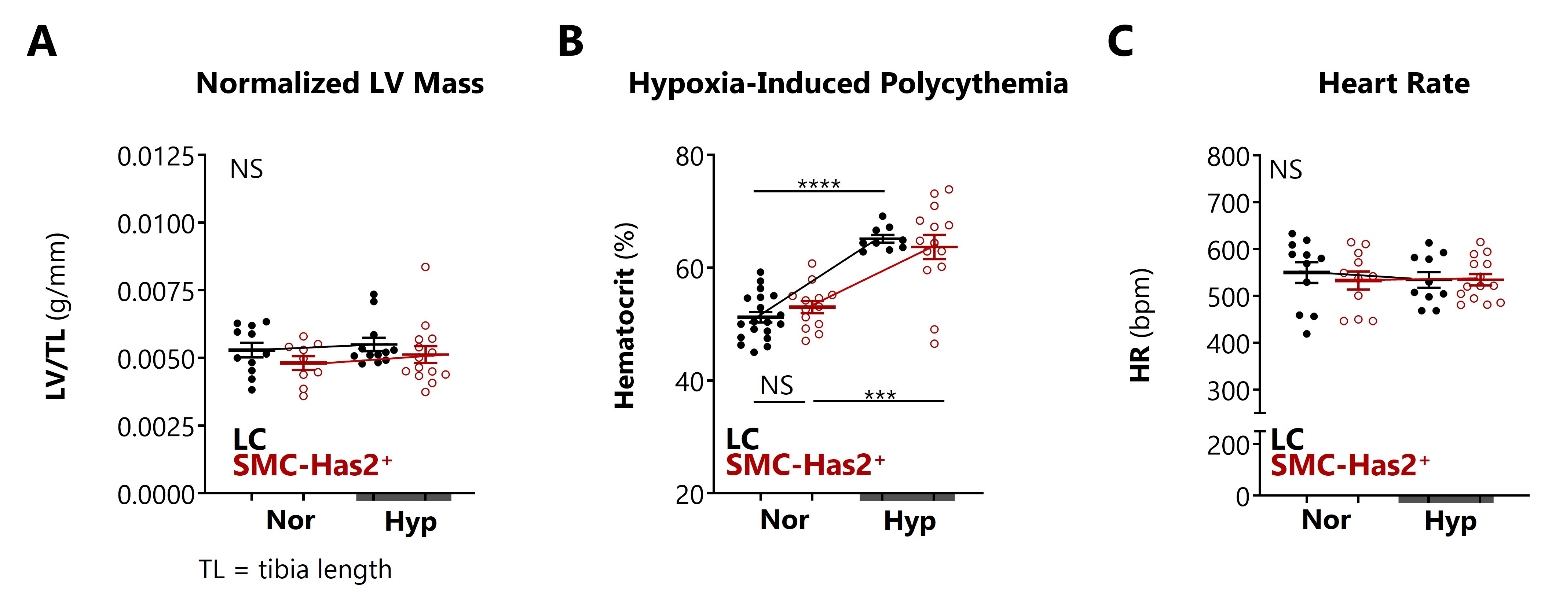
**

**SUPPLEMENTAL FIGURE S5**

**
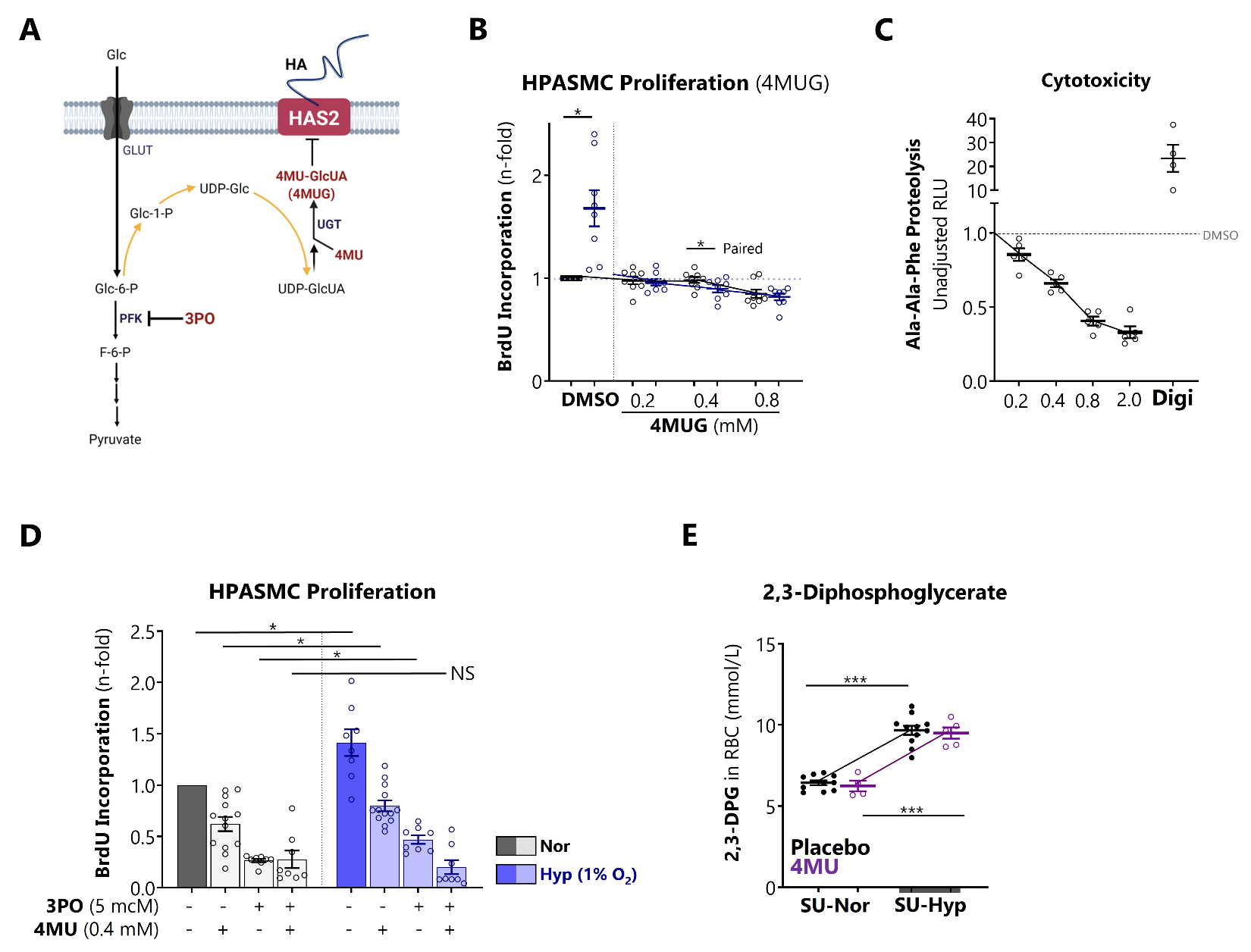
**

**SUPPLEMENTAL FIGURE S6**

**
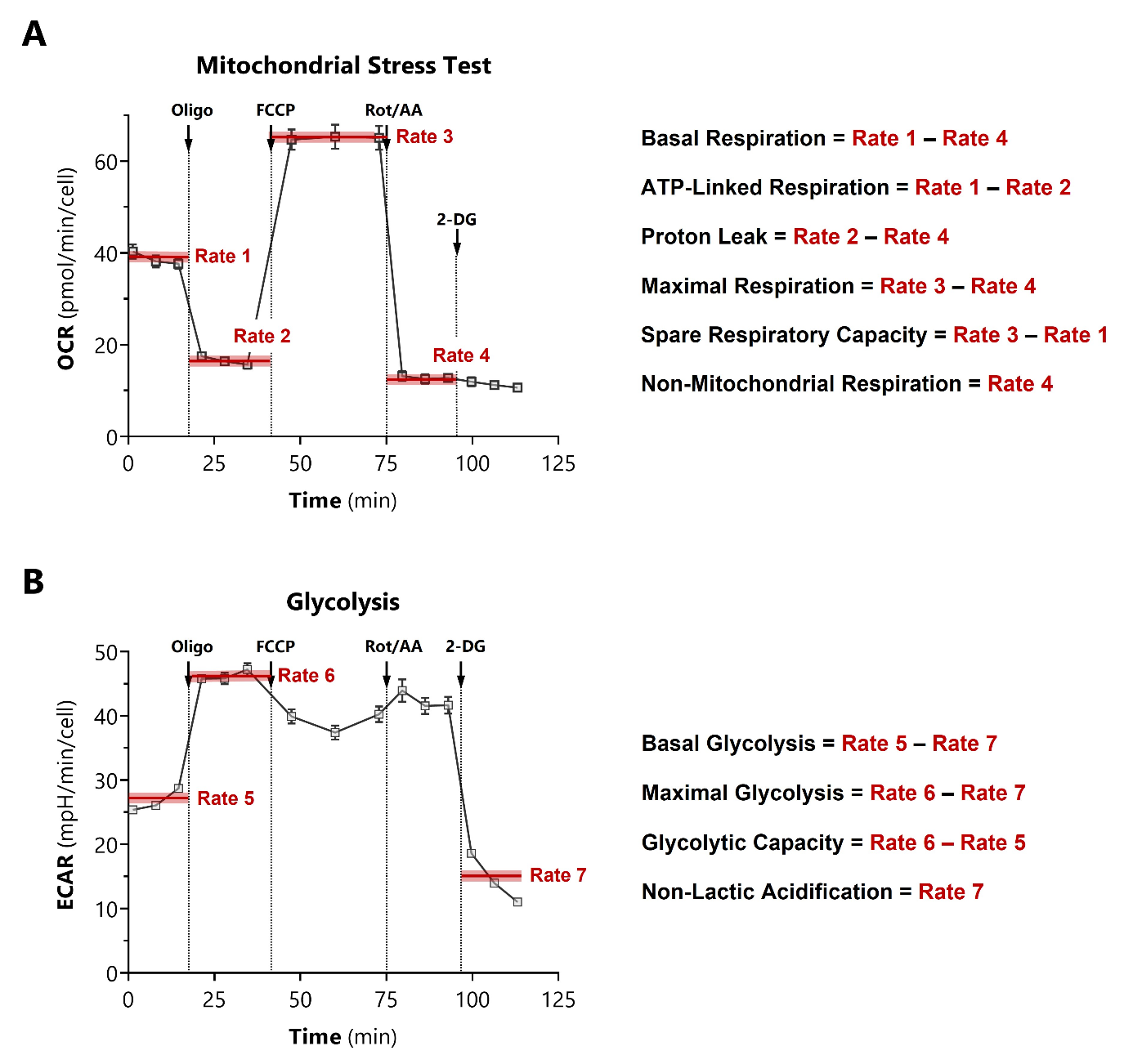
**

**SUPPLEMENTAL FIGURE S7**

**
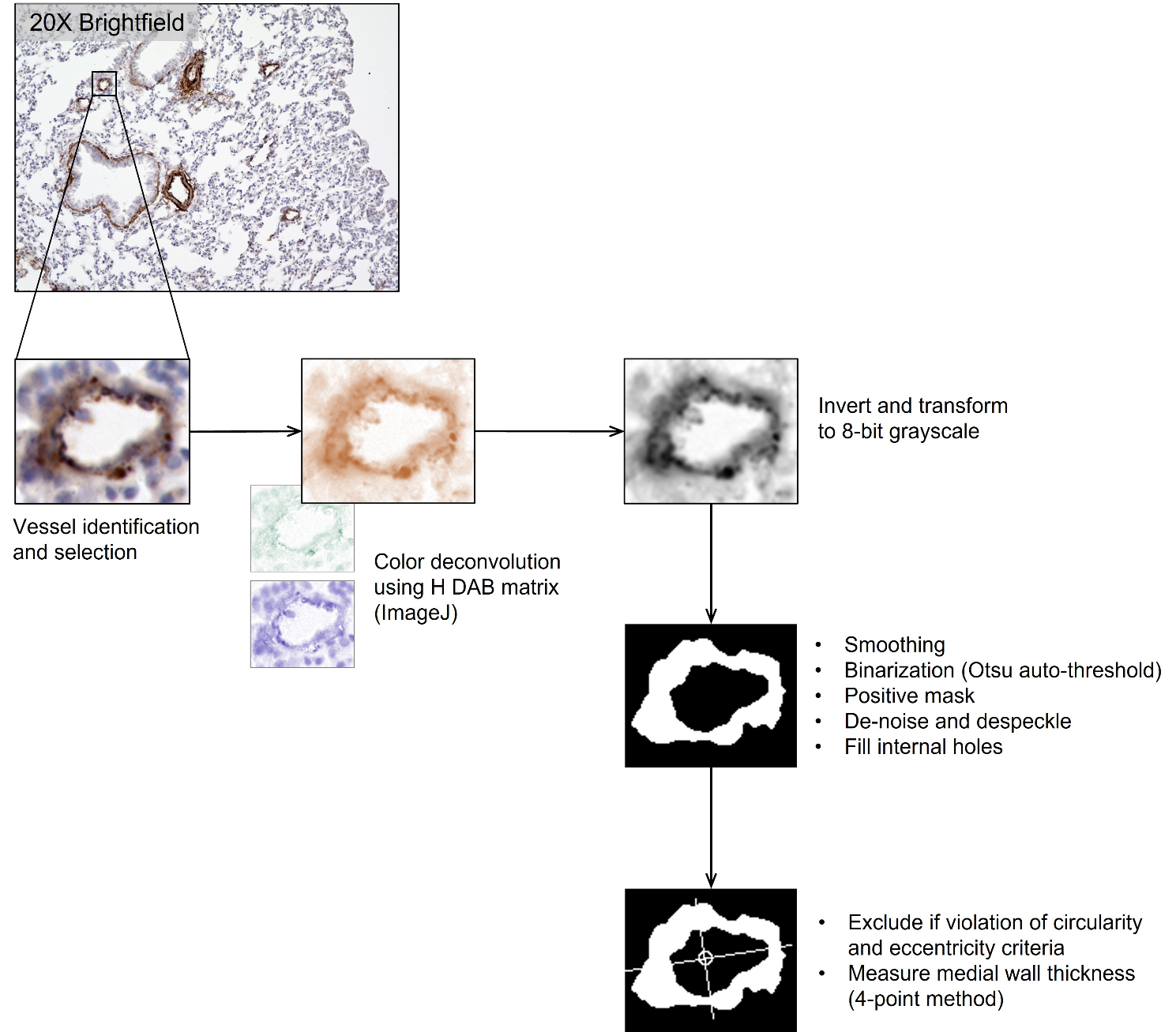
SUPPLEMENTAL FIGURE S8**
