# Supplementary material for: 3’UTR shortening of HAS2 promotes hyaluronan hyper-synthesis and bioenergetic dysfunction in pulmonary hypertension
Source: Matrix Biol. Author manuscript; Available in PMC 2023 Aug 1. (PMC9676077; doi:10.1016/j.matbio.2022.06.001)
Supplement: Supplementary materials 2 [file NIHMS1849092-supplement-Supplementary_materials_2.docx]

**ONLINE SUPPLEMENTAL MATERIALS**

1. Supplemental Figures Legends
2. Supplemental Figures S1 – S8
3. Detailed Methods

**SUPPLEMENTAL FIGURE LEGENDS**

**Supplemental Figure S1**. **Cell-specific production of pulmonary HA**.

**A**: Flow gating strategy for detection of surface HAS2 expression in lung cells from mice with chronic hypoxia-induced PH.

**B**: Production of HA by non-SMC lung cell types cultured in normoxia vs hypoxia (1% O_2_) for 72 hours.

Data expressed as mean ± SEM. *p < 0.05 by 1-way ANOVA by normoxia vs hypoxia.

**Supplemental Figure S2. Quantitative analysis of HPASMC bioenergetic indices.**

**A – H**: Quantification of the (A) basal, (B) ATP-linked, (C) maximal, and (D) spare capacity of mitochondrial oxidative respiration; (E) basal, (F) maximal, (G) oligomycin-induced increase, and (H) reserve capacity of glycolysis in HPASMCs treated with various preparations of HA. Interestingly, the effect of HA provisioned as a substrate coating (HA-C) induced a different bioenergetic profile than soluble HA added to the media; HA-C alone promoted glycolytic metabolism, HA-C combined with HMWHA enhanced glycolytic reserve, but these conditions had minimal impact on OCR.

**I – L**: Quantification of (I) basal, (J) maximal, and (K) reserve capacity of glycolysis in HPASMCs treated with PDGF-BB (50 nM) and 4MU. (L) Proliferation of HPASMCs treated with PDGF-BB and the glycolytic inhibitor 3PO (0.5 micromolar).

Data expressed as mean ± SEM. *p < 0.05, **p < 0.01, ***p < 0.001, and ***p < 0.0001 by 1-way ANOVA by treatment group (A – H), and 2-way ANOVA by [PDGF x 3PO] (I – K) or [PDGF x 3PO] (L).

**Supplemental Figure S3. Impact of acute hyaluronan recycling on HPASMC bioenergetics**

**A, B**: Extracellular acidification rate (ECAR) and; (B) Representative HPASMC oxygen consumption rate (OCR) profiles in cells transduced with adenovirus overexpressing HAS2 (AVCMV-HAS2) versus empty vector (AVCMV-Mock) and treated with recombinant human hyaluronidase PH20 (HYAL) versus heat-killed enzyme as a control (HK-HYAL),

**C**: Determination of key mitochondrial respiratory parameters from OCR profiles, including basal (left panel), ATP-linked (middle panel), and maximal (right panel) respiration.

Data expressed as mean ± 95% CI (A) or mean ± SEM (B, C). *p < 0.05 by 2-way ANOVA of [AV-HAS2 x HYAL] interaction (C)

**Supplemental Figure S4**. **Representative force tracings of pulmonary artery contractile and vasorelaxant stimuli**

**A**: Experimental schematic for *ex vivo* vessel overexpression or depletion of HA. Intralobar resistance PA segments were isolated from control (SU-NOR) or PH (SU-HYP rats). Vessels from SU-NOR control rats were transduced overnight with a high titer (50 million PFU per artery) of AdCMV-HAS2 versus mock (Ad^CMV^-Empty) control adenovirus to acutely increase HA synthesis, mimicking the raised levels observed in PH. To acutely deplete HA from normal or diseased vessels, PA segments from SU-NOR and SU-HYP PH rats were treated with recombinant human PH-20 hyaluronidase (rHYAL) for 4 hours (1.6 U/mL) to denude HA.

**B**: SU-HYP rats developed increased RVSP as expected

**C**: Confirmation of HA depletion in pulmonary artery rings following enzymatic digestion with hyaluronidase.

**D**: Representative force myographs showing single-vessel isometric tension responses for KCl contraction, phenylephrine (PhE) contraction, methacholine (MCh) relaxation, and sodium nitroprusside (SNP) relaxation in rat PA segments treated with Ad^CMV^-HAS2 or rHYAL to overexpress and deplete HA respectively.

**Supplemental Figure S5 Additional characterization of smooth muscle-targeted HAS2 overexpressing mice**

**A:** Combined left ventricular and septal (LV+S) mass normalized to tibial length revealed no differences by strain or chronic hypoxia-induced PH

**B:** Hematocrit, measured by the microcapillary tube method, revealed no differences by strain or chronic hypoxia-induced PH

**C:** Heart rate, measured during right ventricular catheterization via internal jugular vein, revealed no differences by strain or chronic hypoxia-induced PH

Data expressed as mean ± SEM. ***p < 0.001 and ****p < 0.0001 by 2-way ANOVA of [genotype x oxygen group].

**Supplemental Figure S6. Investigation of antiproliferative mechanisms of 4MU on hypoxic and IPAH-derived HPASMCs.** Since the maximal utility of anti-remodeling drug therapy in PH requires hypoxic chemoresistance to be overcome, we leveraged known features of 4MU metabolism to enhance its efficacy in hypoxic conditions. Hypoxia decreases the rate of substrate glucuronidation, and 4MU is one of a few compounds that is activated rather than eliminated by glucuronidation[70, 73]. We therefore treated HPASMC with 4MU glucuronide (4MUG) to bypass the hypoxic block in glucuronidation.

**A**: Schematic depiction of the intersection between glycolytic and glucuronide synthesis pathways showing the sites of inhibition by 3PO, 4MU, and 4MUG.

**B**: Dose-dependent effect of 4MUG on HPASMC proliferation. As expected, 4MUG resulted in equal or greater antiproliferative effects on hypoxic HPASMCs. However, the magnitude of effect was small relative to 4MU, likely because glucuronidation of 4MU rendered it cell-impermeable. Loss of glucuronidation capacity in hypoxia is due to acceleration of glycolysis stealing glucose-6-phosphate (G6P) away from the uronic acid pathway[81]. To maintain flux through the glucuronic acid pathway, we blocked glycolysis by inhibiting phosphofructokinase-2 with 3-(3-Pyridinyl)-1-(4-pyridinyl)-2-propen-1-one (3PO)[82], immediately downstream of G6P.

**C**: Verification that 4MU does not induce cytotoxicity to HPASMCs using the MultiTox cell death assay. Digitonin (Dig, 2%) was used as a positive control for toxicity. Importantly, we found no evidence that 4MU induced cytotoxicity in HPASMCs, providing assurance that the antiproliferation was not merely a nonspecific response of impaired survival. These findings highlight multiple new metabolic targets relevant to the regulation and control of HPASMC proliferation.

**D**: Effect of simultaneous inhibition of glycolysis with 3PO and HA synthesis with 4MU. As expected, 3PO inhibited normoxic proliferation, whereas hypoxia induced resistance. However, we observed that 3PO restored the antiproliferative potency of 4MU in hypoxic conditions.

**E**: Verification that 4MU did not interfere with 2,3-diphosphoglycerate (2,3-DPG) synthesis in erythrocytes, which can alter oxyhemoglobin affinity.

Data expressed as mean ± SEM. *p < 0.05, **p < 0.01, and ***p < 0.001 by 2-way ANOVA of [4MUG dose x oxygen group] (B) or [4MU x PH group] (E), and 3-way ANOVA of [3PO x 4MU] x [Normoxia x Hypoxia] (D).

**Supplemental Figure S7**. **Analysis of Seahorse XF bioenergetic assay results**.

**A**: Representative tracing of oxygen consumption rate (OCR) during the mitochondrial stress test.

**B**: Representative tracing of extracellular acidification rate (ECAR) during the glycolysis assay

Oligomycin (‘Oligo’) inhibits ATP synthase. FCCP dissipates the intermembrane proton gradient, uncoupling mitochondria to produce maximal respiration. Rotenone and Antimycin A (‘Rot/AA’) inhibit Complex I and cytochrome c oxidase (Complex 3) of the electron transport chain, respectively. 2-deoxyglucose inhibits hexokinase, the first committed step of glycolysis.

**Supplemental Figure S8**. **Image processing algorithm for measurement of fractional medial wall thickness from colorimetric immunohistochemistry**. Vessels were visually adjudicated and cropped from 20X magnification images. The α-SMA signal was obtained by color deconvolution along the H 3,3’-diaminobenzadine matrix. A threshold was applied to generate binary masks for each vessel. The medial wall area (MWA) was calculated using the BoneJ plugin available in ImageJ.

**SUPPLEMENTAL METHODS**

**Reagents and Materials**

| **Rodents** | | | | | |  |
| --- | --- | --- | --- | --- | --- | --- |
| Species | Vendor or Source | Background | Sex | | Comments | |
| *Mus musculus* | JAX | C57BL/6J | M/F | | Stock No. 000664 | |
| *Mus musculus* | Lise Wogensen Bach  (Aarhus University) | C57BL/6J | M/F | | αSMA-Has2^+^ | |
| *Mus musculus* | JAX Stock No: 006878 | C57BL/6J | M/F | | SM22-Cre Ki to generate SM22-Has2^KO^ | |
|  | Yu Yamaguchi  (Sanford Burnham Prebys) | C57BL/6J | M/F | | SM22-Has2^Flox^ | |
| *Rattus norvegicus* | Charles River | Sprague Dawley | M | | Strain Code 400 | |
| **Cultured Cells** | | | | | |  |
| Name | Source | Attributes | | Demographic Information | |  |
| PHBI-ST-010 | Pulmonary Hypertension Breakthrough Initiative | IPAH  PASMC | | M (24) | |  |
| PHBI-CC-014 |  |  |  | M (45) | |  |
| PHBI-CC-016 |  |  |  | M (27) | |  |
| PHBI-BA-023 |  |  |  | M (24) | |  |
| PHBI-CC-013 |  |  |  | F (27) | |  |
| PHBI-VA-014 |  |  |  | F (33) | |  |
| PHBI-VA-011 |  |  |  | F (32) | |  |
| PHBI-ST-037 |  |  |  | F (39) | |  |
| PHBI-UA-008 |  | Failed Donor Control (Non-PH) PASMC | | M (25) | |  |
| PHBI-AH-019 |  |  |  | M (52) | |  |
| PHBI-UA-022 |  |  |  | M (26) | |  |
| PHBI-BA-033 |  |  |  | M (24) | |  |
| PHBI-AH-006 |  |  |  | F (28) | |  |
| PHBI-VA-009 |  |  |  | F (52) | |  |
| PHBI-AH-014 |  |  |  | F (28) | |  |
| PHBI-UA-015 |  |  |  | F (36) | |  |
| 3110-0557 | ScienCell | Commercial Control (Non-PH) PASMC | | Unknown | |  |
| 3110-0196 |  |  |  | Unknown | |  |
| 3110-0294 |  |  |  | Unknown | |  |
| 3110-7449 |  |  |  | F (Fetal, 28 weeks) | |  |
| 3110-2828 |  |  |  | Unknown | |  |
| **Antibodies** | | | | | |  |
| Target Antigen | Vendor or Source | Catalog # | | Working Concentration | |  |
| αSMA | Sigma Aldrich (St. Louis, MO) | A5228 | | 1:1000 for IHC | |  |
| αSMA | NeoMarkers (Waltham, MA) | RB-9010 | | 1:1000 for IHC | |  |
| CD44  IgG_2b_ isotype control | Invitrogen (Carlsbad, CA)  R&D Biosystems (Minneapolis, MN) | Clone IM7  MAB0061 | | 2.5 mcg/mL | |  |
| Hyaluronan | CalBioChem (San Diego, CA) | HABP #385911 | | 1:125 for IHC | |  |
| Has2 | Abbexa (Cambridge, UK)  SCBT (Dallas, TX)  MBS (San Diego, CA)  Abcam (Waltham, MA) | ABX10958  clone A-7  MBS9207356  ab140671 (clone E7) | | 1:200 for IHC  1:750 for IB  1:50 for FC  1:1000 for IB | |  |
| NUDT21 | Proteintech (Rosemont, IL) | 10322-1-AP | | 1:100 for IHC  1:1000 for IB | |  |
| CFIm59 | Proteintech (Rosemont, IL) | 55195-1-AP | | 1:1000 for IB | |  |
| CFIm68 | Proteintech (Rosemont, IL) | 15489-1-AP | | 1:1000 for IB | |  |
| CPS73 | Proteintech (Rosemont, IL) | 11609-1-AP | | 1:1000 for IB | |  |
| SM22a-AF405 | Abnova (Taipei, TW) | Clone SPM606 | | 1 mcg/10^6^ cells for FC | |  |
| CD31-BUV395 | BD BioSciences (Franklin Lakes, NJ) | Clone MEC 13.3 | | 1:50 for FC | |  |
| CD140a-BB700 | EMD Millipore (Burlingame, MA) | Clone APA5 | | 1 mcg/10^6^ cells for FC | |  |
| CD45-PE | Invitrogen | Clone 30-F11 | | 0.5 mcg/10^6^ cells for FC | |  |
| GAPDH | Themo Fisher (Waltham, MA)  CellSignaling (Danvers, MA) | AM4300  CST2118S | | 1:1000 for IB  1:2000 for IB | |  |
| β-Actin | Sigma Aldrich | A5316 | | 1:1000 for IB | |  |
| **Oligonucleotides and other sequence-based reagents** | | | | | |  |
| Name | | Sequence | | | |  |
| *hs*HAS2 Distal Forward | | Ctggcagtgttttcccagaac | | | |  |
| *hs*HAS2 Distal Reverse | | Caggccacagaacaaaacct | | | |  |
| *hs*HAS2 Total Forward | | cagtgctctgaaggctgtgt | | | |  |
| *hs*HAS2 Total Reverse | | Tcccggtgagacagatgagt | | | |  |
| *hs*HAS1 Forward | | GAGCCTCTTCGCGTACCTG | | | |  |
| *hs*HAS1 Reverse | | CCTCCTGGTAGGCGGAGAT | | | |  |
| *hs*HAS3 Forward | | CGCAGCAACTTCCATGAGG | | | |  |
| *hs*HAS3 Reverse | | AGTCGCACACCTGGATGTAGT | | | |  |
| *hs*NUDT21 Forward | | tgaagttgaaggactaaaacgct | | | |  |
| *hs*NUDT21 Reverse | | accagttaccaatgcaatcgtc | | | |  |
| *ms*Has2 Forward | | GAGGAAGACCCTATGGTTGGA | | | |  |
| *ms*Has2 Reverse | | GGAAGGAGATCCAGGAATCA | | | |  |
| *ms*Has1 Forward | | GCATGGGCTATGCTACCAA | | | |  |
| *ms*Has1 Reverse | | TCAACCAACGAAGGAAGGAG | | | |  |
| *ms*Has3 Forward | | CTTGGAAGAAGATCCCCAAGT | | | |  |
| *ms*Has3 Reverse | | TCCATGAATCATACTTGTTGAGGA | | | |  |
| NUDT21 siRNA | | SASI_HS01_00146877  Sigma MISSION siRNA; 50 ng/mL | | | |  |
| HAS2 siRNA | | DsiRNA hs.Ri.HAS2.13.2 (NM_005328 Exon 2)  (IDT; Coralville, IA; 50 nM) | | | |  |
| **Chemicals, Enzymes and Other Key Reagents** | | | | | |  |
| Reagent | Description | Vendor or Source | | Catalog # | |  |
| 3-(3-Pyridinyl)-1-(4-pyridinyl)-2-propen-1-one (3PO) | Glycolytic inhibitor of fructose-2,6-bisphosphate 3 (PFKFB3) | Cayman Chemicals (Ann Arbor, MI) | | 19276 | |  |
| 4-methylumbelliferone (4MU) | Competitive inhibitor if HA synthases | Sigma (St. Louis, MO) | | M1381 | |  |
| Carbonyl cyanide *m*-chlorophenyl hydrazone | Mitochondrial electron transport chain uncoupler | Cayman Chemicals | | 25458 | |  |
| High molecular weight hyaluronan (HMWHA) | Mean MW = 1 megadalton | LifeCore Biomedical (Chaska, MN) | | HA-1M | |  |
| Methacholine chloride | Endothelium-dependent vasorelaxant | Sigma | | A2251 | |  |
| Phenylephrine hydrochloride | α_1_ agonist vasoconstrictor | Sigma | | 1533002 | |  |
| Recombinant human PH20 hyaluronidase | EC 3.2.1.35 hyaluronoglucosaminidase | Creative Enzymes (Shirley, NY) | | NATE-1923 | |  |
| Recombinant human platelet-derived growth factor-BB | Pulmonary artery smooth muscle mitogen | R&D Systems (Minneapolis, MN) | | 220-BB | |  |
| Sodium nitroprusside | Endothelium-independent vasorelaxant | Sigma | | 1614501 | |  |
| *Streptomyces hyalurolyticus* hyaluronidase | EC 4.2.2.1 | Seikagaku-Sigma | | H1136 | |  |
| Sugen-5416 (Semaxinib) | VEGFR-2 kinase inhibitor for indication of rodent PH | Tocris (Minneapolis, MN) | | 3037 | |  |

**Human pulmonary artery isolation.** Pulmonary arteries (PAs) were identified visually and dissected free from explanted lung tissue, beginning with a midline transverse cut, separating proximal and distal sections for each lobe. Isolated arterial segments ranged in diameter from 0.5 to 4 mm. The PAs were flash frozen in liquid nitrogen and pulverized using the 6875 CryoMill (SPEX SamplePrep). Demographic and hemodynamic data are summarized in the table below:

| **Human Pulmonary Artery Samples** | | |
| --- | --- | --- |
|  | Failed Donor Control  (*n* = 5) | PAH  (*n* = 5) |
| Age | 30.8±7.9 | 44.6±16.9 |
| Females | 2 | 5 |
| BMI | 26.4±4.6 | 26.4±5.4 |
| Systolic PAP | NA | 79.4±7.8 |
| Diastolic PAP | NA | 38.6±7.3 |
| mPAP | NA | 49.6±6.9 |

**Rodents.** Male and female C57Bl/6J mice (4-12 weeks old, colonies maintained in our animal facilities) or male Sprague-Dawley rats (200-250 gm, Charles River) were used for all experiments. SMC-targeted HAS2 transgene overexpressing (SMC-HAS2^+^) mice[39] were obtained as a gift from Dr. Lise Wogensen Bach (Aarhus University, Denmark). The mice overexpress full-length human HAS2 under the SMP8 alpha-SMA promoter. Mice were previously backcrossed for 14 generations against a C57Bl/6J wildtype (WT) background; heterozygotes and their littermate controls were used for experiments. The SMC-targeted HAS2 knockout (SMC-HAS2^KO^) mice were generated by crossing HAS2^Flox^ mice, a gift from Dr. Yu Yamaguchi (Sanford Burnham), with Sm22/Transgelin-Cre mice. To ensure maximal blinding, mice were randomly assigned to experimental arms and genotyped after sacrifice. Mice were assessed twice weekly for changes in body weight, lack of grooming or hunched posture by lab personnel and weekly by veterinarians at our facilities. Mice were housed socially with a standard 12-hour light/dark cycle and fed standard chow and water *ad libitum*.

**Rodent PH Models**. *Chronic Hypoxia and Sugen-Hypoxia (SU-HYP) PH Models*: Mice were exposed to chronic isobaric hypoxia for 14 and 28 days. Briefly, animals in open cages were placed in isobaric plastic chambers (Biospherix A-chamber; Lacona, NY) and exposed to inspired O_2_ fraction of 10%. The oxygen levels were maintained using an atmospheric regulator from OKO Labs (Pozzuoli, NA, Italy). The vascular endothelial growth factor receptor antagonist, Sugen5416 (20 mg/kg) was injected IP once-weekly in mice during exposure to 10% oxygen for 14 or 28 days. Rats received a single IP injection of Sugen5416 (20 mg/kg) followed by exposure to 10% oxygen for 3 weeks followed by a return to normoxia for an additional 2 weeks. After assessment of PH as described below, rodents were euthanized by isoflurane overdose followed by cervical dislocation and bilateral thoracotomy. Lungs were perfused through the RV and the left lobe excised for histology.

**4MU Treatment for PH Mitigation**. SU-HYP PH was induced in mice as described above. Subsequently, 4MU (125 mg·kg^−1^) medicated chow or control chow (Teklad Industries, Indianapolis, IN, USA) was supplied starting on day 14 and maintained for the remainder of the experiment as described previously[5, 42] .

**Assessment of Pulmonary Hypertension**. Right ventricular systolic pressure (RVSP) was determined by right internal jugular vein cannulation in SMC-HAS2^+^ mouse and SU-HYP rat cohorts as described previously[83]. In SMC-HAS2^KO^ and 4MU mouse cohorts, RVSP was performed by transdiaphragmatic cardiac puncture as previously described[84]. Data were captured in LabChart (AD Instruments; Colorado Springs, CO). A 30-second interval comprising eligible parameters for HR (400 – 500 bpm), end-diastolic pressure (≤ 5 mmHg), and systolic variation (<10%) were selected for analysis. RVSP measurements were excluded if readings fell outside of these limits. RV hypertrophy (RVH) was reported as Fulton’s Index (wet weight ratio of RV/[LV+Septum]).

**Determination of HA Content**. HA concentrations were assayed using the Aggrecan HA-binding protein (HABP) G1 Link-domain based ELISA-like assay (Quantikine, R&D Systems; Minneapolis, MN). The ELISA was loaded with 1 µg sample protein and the assay was performed according to the manufacturer’s protocol. Optical density was taken at 450 nm (colorimetric signal) and 540 nm (background) in a standard plate reader (Omega, BMG LabTech; Ortenberg, Germany). Sample concentrations were fit to the standard curve generated by 4-parameter logistic (4PL) regression (ELISAKit software, Melbourne, Australia). Samples with OD exceeding the upper limit of linearity were diluted and repeated.

**Flow Cytometry Quantitation of Surface HAS2**. Whole lung single cell suspensions were prepared and analyzed by flow cytometry. One mouse lung was gently minced using a GentleMACS C tube (Miltenyi Biotech, Auburn, CA) using Liberase^TM^ at a final concentration of 0.4mg/ml in Ca^2+^/Mg^2+^-free HBSS. After digestion, RBC lysis was performed using RBC lysis buffer (ThermoFisher, Waltham, MA). Single cell suspensions were generated by passing cell suspensions through a 70 micron mesh followed by a 40 micron mesh. FC receptors were then blocked using anti-CD16/CD32 antibody (BD Biosciences, San Jose, CA). Rabbit anti-mouse HAS2 antibody (#MBS9207356) was conjugated to Alexa Fluor 647 using the Lightning-Link kit (Expedeon, San Diego, CA). Cell surface staining was performed using HAS2-AF647, CD45-PE (Clone 30-F11), CD31-BUV395 (Clone MEC 13.3), and CD140a-BB700 (Clone APA5) antibodies. Cells were then fixed and permeabilized using a Cytofix/Cytoperm kit (BD Biosciences, San Jose, CA) per manufacturer guidelines and stained with SM22a-AF405 (Clone SPM606). Cells were analyzed on a ZE5 Cell Analyzer (Propel Labs, Fort Collins, CO). Leukocytes (CD45+) and endothelial cells (CD31+) were first segregated, then smooth muscle cells and fibroblasts (CD140a/PDGFRα+) were segregated. HAS2-AF647 expression was evaluated in each cell population using the mean fluorescence intensity. The gating strategy is detailed in Supplemental Figure **S1A**.

**Analysis of 3’UTR Length**. To detect 3’UTR shortening in HPASMCs, we evaluated distal polyadenylation signal (dPAS) usage by quantitative real-time PCR (RT-qPCR). We used siRNA to silence NUDT21 (MISSION siRNA SASI_HS01_0014687 at 50 ng/mL; Sigma) versus scrambled siRNA for the control condition. Two pairs of primers were designed for HAS2; the first pair targeting the open reading frame to yield the total transcript level, and the second targeting 3’UTR sequences just 5’ of dPAS to detect long transcripts spanning the dPAS. For each gene, the relative abundance of long to total transcripts was quantified as

$$\Delta Ct={Ct}_{distal}-{Ct}_{total}$$

where $Ct$ denotes the PCR cycle threshold. Data were presented as fold change normalized to control using the following computation:

$$\Delta\Delta Ct= \bar{\Delta Ct}_{siCFIm25}-\bar{\Delta CT}_{siScr}={log}_{2}\left( \frac{{DPUI}_{siNUDT21}}{{DPUI}_{siScr}} \right)$$

where $DPUI$ is the distal polyadenylation index. A negative $\Delta\Delta Ct$ value denotes 3’UTR shortening compared to control, with $\left| DPUI \right|>0.2$ considered a significant shift in 3’UTR length[21].

**Immunohistochemistry and Vascular Morphometry**. Formalin-fixed paraffin-embedded lungs were sectioned and processed for immunohistochemistry as previously described[5, 29]. The lung sections were stained for α-SMA and HABP using antibodies listed above.

*Microscopy, Image Processing, and Quantitation of Vascular Remodeling*: For detection of alpha smooth muscle actin (αSMA), mouse lung slides were imaged at 20X brightfield (Keyence BZ-X800; Itasca, IL). HABP was imaged under brightfield or TRITC filter; αSMA was imaged under brightfield or DAPI filter. Mouse lung slides were anonymized and analyzed by a blinded observer. Small pulmonary arteries were identified by visual inspection and cropped. The vessel image was then deconvolved to fetch the 3,3’-diaminobenzadine layer. After converting to 8-bit grayscale, the vessel image was binarized to render a vessel mask. Line integration of the positive pixel count across 360° yielded the total vessel medial wall area (MWA). The fractional MWA relative to total vessel area was determined. We excluded vessels with insufficient circularity ($4\pi\cdot Area/{Perimeter}^{2} <0.6$) from analysis. Our algorithm has been uploaded to the public domain as a modification to the BoneJ plugin of ImageJ. See Supplemental Figure **S8** for a graphical overview.

**Pulmonary Artery Contractility**. Intralobar resistance PA segments were isolated from rats with SU-HYP PH or SU-NOR controls. To acutely digest HA, the vessels were incubated in OptiMEM reduced-serum media (ThermoFisher) with 1.6 turbidity reducing units/mL of *Streptomyces hyalurolyticus* hyaluronidase for 4 hours at 37°C. For control treatments, the hyaluronidase was heat-inactivated at 95°C for 15 minutes. At least 90% clearance of HA was confirmed by ELISA. To overexpress HA, the vessels were infected with 10^7^ PFU of adenovirus, Ad^CMV^-huHAS2 (Vector Bio Labs; Malvern, PA) for 24 hours in OptiMEM supplemented with 10 mM glucose to enhance HA production rate. Effective HA overexpression was similarly confirmed by ELISA.

Pulmonary artery rings were prepared and their contractile properties were assessed as previously reported[85]. Briefly, the rings were washed in PBS and mounted on differential capacitance dynamometers (Harvard Apparatus; Holliston, MA) in isolated tissue baths containing Krebs-Henseleit buffer (130 mM sodium chloride, 2.5 mM potassium chloride, 25 mM sodium bicarbonate, 1.2 mM sodium phosphate, 1.2 mM magnesium chloride, 2.5 calcium chloride, 7.5 mM D-glucose). The muscle baths were aerated with oxygen, and after equilibration, the vascular ring passive tension was set at 10 mN. Rings were contracted with a gradient of potassium chloride (5 – 80 mM) as a positive control test for constriction. Next, the sensitivity to alpha adrenergic contraction was tested using a logarithmic gradient of phenylephrine (10^-9^ to 3 x 10^-5^ M). Next, rings pre-contracted to 80% maximal tension with phenylephrine were treated with a logarithmic gradient of methacholine (10^-9^ to 3 x 10^-5^ M) to assess endothelium-dependent, agonist-induced vasodilation. Sodium nitroprusside was used as a positive control for relaxation. Data were recorded into LabChart (AD Instruments), and adynamic vessels (< 25% contraction and/or < 25% relaxation) were assumed to be damaged and were excluded from analysis. The EC50 for each vessel was calculated from the logistic best-fit Hill equation, where $F_{d}$ is the absolute force at agonist dose$d$:

$$F_{d}=F_{Baseline}+ \frac{F_{Max}-F_{Baseline}}{1+ \left( \frac{d}{EC50} \right)^{H}}$$

**Cell Culture.** Individual primary HPASMC lines (ScienCell; Carlsbad, CA) were confirmed to be genetically unique using short tandem repeat (STR) analysis. Cells were cultured in smooth muscle growth media (SmGM-2; Lonza) supplemented with penicillin/streptomycin, 2% fetal bovine serum (FBS), and smooth muscle growth supplement (SMGS, Lonza). Control and PAH-derived HPAMSCs lines were obtained from the PH Breakthrough Initiative[86]. Normal human pulmonary artery endothelial cells (HPAEC, Invitrogen clones 1621528 and 631255) were cultured in endothelial growth medium (EGM2, Lonza) supplemented with SingleQuots growth factors and 5% FBS. Normal human lung fibroblasts (NHLF, ATCC) were cultured in F12K media (ATCC) supplemented with 2 mM L-glutamine and 10% FBS. Human bronchial epithelial cells (BEAS-2B, ATCC) were cultured in BEGM media (Lonza) supplemented with 10% FBS on dishes coated with collagen-fibronectin-BSA matrix. Cells between passages 4 and 8 were used in all experiments.

**Cell Hypoxia Exposure**. Selected cells were exposed to hypoxia (1% O_2_ for 72 hours) using a continuous O_2_ controller (OKOlab; Pozzuoli, Italy). To avoid re-oxygenation, all hypoxic cell manipulations were performed in a closed modular hypoxia glovebox.

**Manipulation of HA**: HPASMCs were treated with 4MU (Sigma, 0.2-0.8 mM) for 24-72 hours to deplete HA. To silence has2, the cells were transfected with Dicer-substrate DsiRNA (NM_005328 Exon 2; Integrated DNA Technologies; Coralville, IA) at 10-50 nM using RNAiMAX Lipofectamine (ThermoFisher) in OptiMEM for 4 hours. Knockdown was confirmed by immunoblotting and ELISA for HA. To lyse extracellular HA, the cells were treated with 3.125-12.5 turbidity reducing unit of *S. hyalurolyticus* hyaluronodinase (Seigigaku; Tokyo, Japan) or recombinant human PH20 HYAL for 24-72 hours. To simulate HA overload, cells treated with exogenous 1.5 megadalton endotoxin-free high molecular weight HA (HMWHA; LifeCore HA; Chaska, MN). In some experiments, cells were grown on HA-coated plates (25-100 mcg HMWHA per cm^2^) prepared by adsorbing HA onto polystyrene overnight at 50°C. To increase endogenous production of HA, HPASMCs were transduced with adenovirus, Ad^CMV^-huHAS2 vs Ad^CMV^-Empty or Ad^CMV^-GFP. To interrupt HA signaling through CD44, cells were treated with rat anti-human CD44 mAb (clone IM7; Invitrogen, 2.5 mcg/mL) or its IgG_2b_ isotype control (R&D Biosystems; clone MAB0061). To block PFK-2 and inhibit glycolysis, cells were treated with 3-(3-Pyridinyl)-1-(4-pyridinyl)-2-propen-1-one (3PO, Cayman Chemicals, 2 μM).

**Measurement of Proliferation.** The proliferation of HPASMCs was assessed by rate of incorporation of 5-bromo-2’-deoxyuridine (BrdU, EMD Millipore) as a reporter of *de novo* DNA synthesis. Briefly, cells were seeded at low density (400-800 cells/well) into 96-well plates and allowed to adhere overnight. The BrdU label was added to the cells for at least 6 hours. The cells were fixed for 30 minutes with formalin and stored overnight at 4°C. Cell labeling was detected with ELISA utilizing mouse anti-BrdU primary antibody and HRP-goat anti-mouse secondary antibody. Colorimetric peroxidase activity was detected with 3,3’,5,5’-tetramethylbenzidine (TMB) substrate, and optical density was determined at 450 nm. In some experiments, proliferation was induced by treating cells with platelet-derived growth factor-BB (PDGF-BB; R&D, 50 nM).

**Measurement of Migration**. A scratch wound migration assay was performed by etching a confluent monolayer of HPASMCs with a Pasteur tip to create a 1 mm linear defect. The media was replaced with DMEM containing low serum (0.1% FBS) and 1 μM Mitomycin-C to inhibit proliferation. Migration was determined by the number of cells populating the scratch wound at 24 hours.

**Measurement of Apoptosis**: HPASMCs were grown to confluence and treated with 10 mM carbonyl cyanide *m*-chlorophenyl hydrazone (CCCP) for 2 hours to induce acute mitochondrial depolarization and apoptosis. Mitochondrial membrane potential was assessed using MitoCapture[87, 88], a voltage-dependent mitochondrial localized fluorophore (BioVision; Milpitas, CA). The cells were imaged at 20X with TRITC and GFP lasers (Keyence BZ-X800) under oblique illumination and low photobleaching mode. Exposures and gain were titrated to ensure that <0.01% of pixels were zero or saturated. Mitochondrial depolarization resulted in leaching of the red MitoCapture into the cytosol, where depolymerization of the dye resulted in green fluorescence. Changes in mitochondrial polarization were quantified as the ratio of emitted light in the red and green channels (570 and 475 nm, respectively). All images were then randomized and analyzed by a blinded observer.

Apoptosis was also detected by capsase-3 activity, measured by chromogenic cleavage of *p*-nitroanilide-conjugated DEVD peptide (BioVision). Briefly, HPASMC were treated with 20 mcg/mL of HMWHA for 16 hours. Apoptosis induced with CCCP as described above, and staurosporine (1 μM) was used as a positive control. After 2 hours, just prior to morphologic blebbing was apparent, cells were harvested and lysed in protease inhibitor-free buffer. A BCA protein assay was performed and 100 mcg of protein were loaded on a 96-well plate. Dithiothreitol (DTT, 1 mM) and *p*-NA-DEVD (200 μM) were added to the lysate and incubated for 2 hours at 37°C. Absorbance at 405nm was determined by spectrophotometry, and results were expressed relative to uninduced controls.

Finally, apoptosis was confirmed via Annexin-V-FITC and propidium iodide (PI) detection by flow cytometry. After incubation for CCCP or vehicle control, HPASMCs were trypsinized and viable cell counters were collected before proceeding. HPASMCs were rinsed with 1X phosphate-buffered saline (PBS), centrifuged, and resuspended with 500 uL FACS buffer (PBS + 0.01% Sodium Azide + 1% fetal bovine serum). Cells were labeled with 50 mcg/mL of Annexin-V and PI. A BD Accuri C6 Plus Flow Cytometer (Franklin Lakes, NJ) was used to measure cell fluorescence for over 300,000 events. Gating was based on PI and zombie NIR staining, as well as FSC/SSC plots to eliminate debris. Final results show mean Annexin V-FITC fluorescence relative to uninduced controls.

**Assessment of Cytotoxicity**. The cytotoxicity of various experimental treatments was assessed using the MultiTox assay (Promega; Madison, WI). Briefly, treated cells were incubated with an aminoluciferin-conjugated tripeptide (AFC-alanyl-alanyl-phenylalanine). Upon cell death, proteolytic release of AFC enabled kinetic luminescence to be measured after addition of recombinant luciferase. 2% digitonin was used as a positive control.

**Cellular Bioenergetics.** HPASMCs were seeded into a Seahorse Extracellular Flux Analyzer 96-well cell culture plate (Agilent; Santa Clara, CA) pre-coated with 20 mcg/cm^2^ fibronectin and grown to 75% confluence in the presence of treatments enumerated above. The growth medium was replaced with XF assay media containing 1 mM sodium pyruvate, 1 mM L-glutamine, and 10 mM D-glucose titrated to a pH of 7.45. The media was decarboxylated for 30 minutes in a CO_2_-less incubator. The cell plate was mated with O_2_ and pH-sensing electrodes to measure oxygen consumption rate (OCR) and extracellular acidification rate (ECAR), respectively. Baseline OCR and ECAR were measured, followed by sequential injections of oligomycin (1 μM), FCCP (1 μM), rotenone/antimycin A (0.5 μM) and 2-deoxyglucose (50 mM) to sequentially inhibit components of the electron transport chain and glycolysis. Basal, ATP-linked, maximal, spare capacity, non-mitochondrial respiration, and total glycolytic capacity were calculated from these OCR and ECAR profiles as detailed in Supplemental Figure **S7**. For cell energy phenotype testing, OCR and ECAR were measured before and after injection of an acute stressor mix consisting of oligomycin (1.5 μM) and FCCP (1.25 μM). To account for potential differences in proliferation or cell detachment, all bioenergetics values were normalized to cell count or total protein recovered per well.

**Mitochondrial Copy Number**. Total DNA was extracted from HPASMCs using DNeasy spin columns (QIAGEN; Germantown, MD) according to the manufacturer protocol. Real-time qPCR was performed on 2 ng of total DNA, with primers against two nuclear encoded genes, *BECN1* and *NEB1,* and against two mitochondrially encoded genes *ND1* and *ND6*. Rho(0) DNA was used as a negative control. Relative mitochondrial copy number was determined by the average ΔCT ratio between *BECN1*/*ND1* and *NEB1*/*ND6*. The results were further confirmed using the standard curve method, generated with a positive control sample of 143B DNA.

**RT-qPCR**. Flash frozen lungs were pulverized with a mortar and pestle. RNA was extracted with Triazole and chloroform. RNA was obtained by addition of Cotrimoxazole lysis reagent (QIAzole, QIAGEN; Hilden, Germany) followed by extraction with chloroform added in a 1:5 ratio. Following precipitation with 100% ethanol, samples were passed through an RNA-binding spin column (miRNeasy QIAGEN). The column was washed and mRNA eluted with water. Total RNA was quantified by A260/280 ratio via NanoDrop. cDNA synthesis was performed by reverse transcription reaction (iScript, BioRad) using 2000 ng of template. For RT-qPCR reactions, between 20 – 40 ng of cDNA were utilized per well and amplified with Sybr-Green primer pairs listed above. No-template controls were included in each experiment. Relative expression (Rq) against the housekeeping gene *9S* for mouse samples or *18S* ribosomal RNA in HPASMCs was determined via the double delta C_t_ method. Fluorometric amplification signal was monitored in real time with an ABI 7500 Real-time PCR system (Applied Biosystems; Foster City, CA). Replicates were included only if the standard deviation of C_t_ values was < 0.5. If housekeeping C_t_ values were within ±1 cycle, Rq values from multiple experiments were pooled after normalization to wild-type normoxic group mean.

**Immunoblotting**. Proteins were isolated from whole cell lysates by sonicating the cell pellets in a buffer consisting of 20mM Tris (pH 7.4), 2.5mM EDTA, 1% Triton X-100, 1% Deoxycholic acid, 0.1% SDS, 100mM sodium chloride, 10mM sodium fluoride, 1mM sodium orthovanadate (Na_3_VO_4_) supplemented with 1 mM Roche Complete Mini Protease inhibitor and PhosSTOP. Protein was quantified by the BCA method. Between 25-50 mcg of denatured protein were prepared in reducing conditions and loaded onto a 4-15% precast gradient PAGE gel (Criterion Tris-HCl, BioRad). Samples were electrophoresed in MOPS buffer and the protein was then blotted onto nitrocellulose using a wet transfer system (Mini Trans-Blot, BioRad) in cold Towbin buffer (25 mM Tris, 192 mM glycine, and 20% methanol at pH 8.3). The membrane was blocked in 5% milk/TBS-T for 1 hour and probed for Has2 (1:750 SCBT clone A-7; Dallas, TX) and GAPDH (1:2000 CST clone 14C10) overnight at 4°C. The membranes were probed with IRDye 800CW or 680RD IgG secondary antibodies (LI-COR Biosciences; Lincoln, NE) for 1 hour. The bands were visualized on a LI-COR Odyssey NIR scanner (700 and 800 nm) and densitometry was performed in ImageJ.

Pulverized lung or pulmonary artery tissue was dissolved in RIPA buffer (Thermo Scientific, Rockford, IL) containing 1 mM protease and phosphatase inhibitor (Sigma-Aldrich). 20 mcg of protein was mixed with 6X SDS-Sample buffer (Boston BioProducts) and loaded into 4-12% Mini-Protean TGX gels (Bio-Rad, Hercules, CA). These gels were then transferred to PVDF membranes (GE Healthcare, Piscataway, NJ), which were subsequently blocked with 5% milk in TBS-T(Sigma-Aldrich) for 1 hour and incubated with the primary antibody overnight at 4°C. Secondary antibodies were incubated for 1 hour at RT and the blots were visualized with Clarity ECL (Bio-Rad). Densitometries were performed with the Bio-Rad Image Lab software.

References

[81] D.P. Jones, T.Y. Aw, X.Q. Shan, Drug metabolism and toxicity during hypoxia, Drug Metab Rev 20(2-4) (1989) 247-60.

[82] B. Clem, S. Telang, A. Clem, A. Yalcin, J. Meier, A. Simmons, M.A. Rasku, S. Arumugam, W.L. Dean, J. Eaton, A. Lane, J.O. Trent, J. Chesney, Small-molecule inhibition of 6-phosphofructo-2-kinase activity suppresses glycolytic flux and tumor growth, Mol Cancer Ther 7(1) (2008) 110-20.

[83] R.E. Nisbet, J.M. Bland, D.J. Kleinhenz, P.O. Mitchell, E.R. Walp, R.L. Sutliff, C.M. Hart, Rosiglitazone attenuates chronic hypoxia-induced pulmonary hypertension in a mouse model, Am J Respir Cell Mol Biol 42(4) (2010) 482-90.

[84] T.C.J. Mertens, A. Hanmandlu, L. Tu, C. Phan, S.D. Collum, N.Y. Chen, T. Weng, J. Davies, C. Liu, H.K. Eltzschig, S.S.K. Jyothula, K. Rajagopal, Y. Xia, A. Guha, B.A. Bruckner, M.R. Blackburn, C. Guignabert, H. Karmouty-Quintana, Switching-Off Adora2b in Vascular Smooth Muscle Cells Halts the Development of Pulmonary Hypertension, Front Physiol 9 (2018) 555.

[85] J.M. Kleinhenz, T.C. Murphy, A.P. Pokutta-Paskaleva, R.L. Gleason, A.N. Lyle, W.R. Taylor, M.A. Blount, J. Cheng, Q. Yang, R.L. Sutliff, C.M. Hart, Smooth Muscle-Targeted Overexpression of Peroxisome Proliferator Activated Receptor-gamma Disrupts Vascular Wall Structure and Function, PLoS One 10(10) (2015) e0139756.

[86] J.H. Newman, S. Rich, S.H. Abman, J.H. Alexander, J. Barnard, G.J. Beck, R.L. Benza, T.M. Bull, S.Y. Chan, H.J. Chun, D. Doogan, J. Dupuis, S.C. Erzurum, R.P. Frantz, M. Geraci, H. Gillies, M. Gladwin, M.P. Gray, A.R. Hemnes, R.S. Herbst, A.F. Hernandez, N.S. Hill, E.M. Horn, K. Hunter, Z.C. Jing, R. Johns, S. Kaul, S.M. Kawut, T. Lahm, J.A. Leopold, G.D. Lewis, S.C. Mathai, V.V. McLaughlin, E.D. Michelakis, S.D. Nathan, W. Nichols, G. Page, M. Rabinovitch, J. Rich, F. Rischard, S. Rounds, S.J. Shah, V.F. Tapson, N. Lowy, N. Stockbridge, G. Weinmann, L. Xiao, Enhancing Insights into Pulmonary Vascular Disease through a Precision Medicine Approach. A Joint NHLBI-Cardiovascular Medical Research and Education Fund Workshop Report, Am J Respir Crit Care Med 195(12) (2017) 1661-1670.

[87] M. Plataki, S.J. Cho, R.M. Harris, H.R. Huang, H.S. Yun, K.T. Schiffer, H.W. Stout-Delgado, Mitochondrial Dysfunction in Aged Macrophages and Lung during Primary Streptococcus pneumoniae Infection is Improved with Pirfenidone, Sci Rep 9(1) (2019) 971.

[88] H. Okamura, K. Yoshida, B.R. Amorim, T. Haneji, Histone H1.2 is translocated to mitochondria and associates with Bak in bleomycin-induced apoptotic cells, J Cell Biochem 103(5) (2008) 1488-96.
